# Micro Blood Flow-Resolved Rheometry

**DOI:** 10.3390/mi17030331

**Published:** 2026-03-06

**Authors:** Yang Jun Kang

**Affiliations:** Department of Mechanical Engineering, Chosun University, 10, Chosundae 1-gil, Dong-gu, Gwangju 61452, Republic of Korea; yjkang2011@chosun.ac.kr; Tel.: +82-62-230-7052; Fax: +82-62-230-7055

**Keywords:** blood flow-dependent RBC aggregation, blood viscosity, two-step blood delivery, microfluidic chip, blood velocity fields, blood image rate

## Abstract

For effectively assessing blood, red blood cell (RBC) aggregation and blood viscosity have been measured in microfluidic environments. However, the previous methods still face several challenges (dead-volume loss, RBC sedimentation, hematocrit-sensitive blood velocity, and precise flow rate control). In this study, a novel method is suggested to resolve several issues. Air cavity (*V_air_* = 250 μL) is secured above the blood column (at least 100 μL) loaded into a driving syringe. To probe RBC aggregation and blood viscosity, a microfluidic chip consists of a main channel ($\dot{\gamma }$ > 1000 s^−1^) and an aggregation channel ($\dot{\gamma }$ < 50 s^−1^). Blood is supplied into a microfluidic chip with two-step blood delivery (i.e., air compression for RBC aggregation, and syringe pump for blood viscosity). RBC aggregation index and blood viscosity are obtained from time-lapse image intensity and blood flow rate in both channels. As performance demonstrations, first, the measurement accuracy of fluid viscosity is validated with glycerin solution. Then, the present method is adopted to probe the difference in hematocrit and dextran concentration. At last, the proposed method is employed to detect heat-shocked RBCs (45~50 °C for 40 min). In conclusion, the proposed method has the ability to accurately measure substantial changes in RBCs or blood medium.

## 1. Introduction

Blood, as a complex fluid, is composed of cells (i.e., red blood cell [RBC], white blood cell, and platelet) and plasma. In particular, intrinsic properties of RBCs (i.e., membrane viscoelasticity, cytosol viscosity, and morphological parameters) have a strong impact on dynamic blood flows [1,2,3,4]. Plasma protein is also involved in RBC-to-RBC interaction. Several hemorheological properties, including blood viscosity [5,6,7,8,9], RBC aggregation [10,11,12,13,14,15,16], RBC deformability [17,18,19,20,21,22,23,24,25,26], and sedimentation rate, [27,28,29] have been suggested for effectively monitoring physiological alterations in patient blood (i.e., cardiovascular disease [5,6], acute myocardial infarction [11], stroke [12,13], sickle cell anemia [17,18,19,22,30,31,32], and malaria [20]). Among them, under continuous blood flow, blood viscosity has been determined by several factors, such as hematocrit [33], plasma proteins, RBC aggregation at low shears, and RBC deformability at high shears. On the other hand, at stasis or extremely low blood flow, RBC aggregation has been employed to investigate RBC–RBC interaction, which has been strongly influenced by plasma proteins and RBC intrinsic properties.

When compared with conventional methods (i.e., a cone-and-plate viscometer and an ektacytometry [17,18,19]), a microfluidic chip has the ability to probe hemorheological properties with small volume, to control shearing blood-flow precisely, and to provide microvascular-mimicked environments [1,2,16,25,28,30,34,35,36]. Thus, it provides rapid, quantitative, and reproducible multi-parameter readouts [3,22,37] (i.e., blood viscosity [38,39], RBC aggregation, RBC deformability [23,40], RBC sedimentation rate [29,41]).

First, according to the Hagen–Poiseuille law (i.e., pressure difference = fluidic resistance × flow rate) [42], blood viscosity could be estimated by quantifying pressure difference or blood flow rate. Under constant flow rate conditions, blood viscosity could be assessed by monitoring parallel–stream interface in a single channel [43], microflow compartments in multiple channels [8,44], flow-switching in a microfluidic bridge channel, and flow-reversal time in a closed channel [28]. Interface-front tracking is used to estimate blood viscosity under controlled pressure [45,46,47,48,49,50] or capillary force [7,38,51,52]. Blood viscosity could be assessed by a microstructure deflection [53], a resonant-frequency shift [54,55], and droplet kinetics [56].

Second, after aggregated RBCs are fully disaggregated by an external agitator, RBC aggregation could be estimated by analyzing the syllectogram. According to the previous studies [15,57,58,59,60,61,62,63,64], RBC aggregation initiates below $\dot{\gamma }\;$ = 10 or 50 s^−1^. Novel methodologies related to RBC-to-RBC disaggregation agitator (i.e., motor [65,66], pump [15,67], bubble-motion [12,63], and pinch valve [68]) and RBC-to-RBC aggregation quantification (i.e., phototransistor [65,67], microscopic imaging [15], and electrical impedance [36,66]) have been demonstrated to assess RBC aggregation consistently.

Blood viscosity, which has been probed in a microfluidic chip, represents the dominant effect of RBC deformability where shear rate is estimated as a high value of $\dot{\gamma }$ > 10^3^ s^−1^. Blood viscosity is limited at low shear rates. In particular, at low shear rates, RBC aggregation is then quantified independently. Thus, blood viscosity and RBC aggregation have been quantified by stopping and operating two syringe pumps periodically. The previous method requires two bulky syringe pumps and periodic on–off flow rate control [69]. More recently, our group has suggested a new quantification method of RBC aggregation index under continuous blood flow supplied from a single syringe pump [15]. Since the RBC aggregation index is highly dependent on fluidic resistance [15,28,59,62,70,71,72,73,74,75], the previous approach should be substantially refined to enable blood viscosity. The RBC aggregation index should also be represented at shear rates. Furthermore, a non-negligible dead volume (approximately 100 μL) is present along the fluidic path from the syringe to the inlet port [76,77]. Consequently, a portion of blood must be wasted during each run [9,78]. Therefore, a new approach is required to reduce unavoidable blood loss under the specific dead volume in the fluidic path.

In this study, a novel method is suggested to resolve several issues (i.e., dead volume loss, RBC sedimentation, and flow-dependent RBC aggregation, as well as blood viscosity) raised by the previous methods. To reduce blood loss within the fluidic path, the air cavity of *V_air_* = 250 μL is intentionally maintained above the blood column (*V_b_* = 100~200 μL) loaded into the syringe. When air is injected from the syringe into a microfluidic chip, most of the blood initially loaded in the syringe is subsequently delivered into the microfluidic chip. Thus, securing an air cavity in the syringe eliminates unavoidable blood loss in the fluidic path, which reduces the minimum blood loading volume to 100 μL. To eliminate on–off operation of the single syringe pump, the syringe plunger is manually advanced by approximately 50 μL to increase the air pressure inside the syringe. The pressure difference contributes to loading blood into the fluidic path and microfluidic channels. Blood flow decreases gradually over time, which induces RBC aggregation in a microfluidic channel. Based on the blood flow image in a straight main channel (i.e., high shear rates) and a bifurcation channel (i.e., low shear rates), the flow-dependent RBC aggregation index is continuously obtained by calculating the relative ratio of blood image intensity. After an elapse of a certain time, to minimize RBC sedimentation in the driving syringe during blood delivery, the syringe pump is set to a high value of flow rate (*Q_sp_* = 10 mL/h). Due to the compliance effect, the blood velocity rises transiently over time and then reaches a plateau value. When blood velocity is measured by a time-resolved micro particle image velocimetry (PIV), the results are strongly affected by hematocrit. Herein, since the flow rate of the syringe pump and steady-state blood velocity are specified, the blood velocity can be converted into a blood flow rate (i.e., *Q* = *U*/*U_st_* × *Q_sp_*, *U_st_*: steady-state blood velocity, *Q_sp_*: setting flow rate of syringe pump). Air pressure inside the driving syringe is then estimated by analyzing time-lapse blood flow as well as the ideal-gas law (i.e., air volume × air pressure = constant) [50]. Blood viscosity is then determined by the Hagen–Poiseuille law. Thus, RBC aggregation and blood viscosity are obtained during two stages of blood delivery (i.e., air compression and syringe pump). To validate the performance of the suggested method (i.e., RBC aggregation and blood viscosity), first, the viscosity of glycerin solution (*C_gl_* = 20~50%) is obtained. Second, using two kinds of blood medium (i.e., 1× PBS and dextran solution [20 mg/mL]), the effect of RBC sedimentation in the driving syringe is quantified from blood viscosity with respect to delivery flow rate (*Q_sp_*). Third, to find out the effect of hematocrit on RBC aggregation and blood viscosity, the test blood is adjusted to Hct = 30~50% by suspending normal RBCs into dextran solution (20 mg/mL) [65]. Fourth, to induce RBC aggregation to a certain level, test blood is prepared by adding normal RBCs into various concentrations of dextran solution. Fifth, the minimum blood-loading volume into a driving syringe (*V_b_* = 100~200 μL) is evaluated by measuring RBC aggregation and blood viscosity. At last, to investigate biomechanical differences in heat-shocked RBCs, normal RBCs are exposed to 45 °C for up to 40 min and 50 °C for up to 20 min.

Compared to previous methods, first, unavoidable blood loss in the fluidic path is completely eliminated by securing an air cavity (*V_air_* = 250 μL) above the blood column in a driving syringe. Minimum blood loading-volume can be decreased to *V_b_* = 100 μL. Second, test blood is introduced with a hybrid blood delivery platform (i.e., a manual air-compression for RBC aggregation and a syringe pump for blood viscosity). Using bifurcation channels (i.e., main channel: high shear rate, and aggregation channel: low shear rate), blood image intensity at the bifurcation channel can probe the RBC aggregation index at continuously varying shear rates. A non-linear sigmodal regression model (i.e., AI = $\frac{{AI}_{0}}{1+exp(a\left[\dot{\gamma }-b\right])}$) is adopted to conduct quantitative analysis of RBC aggregation. To avoid RBC sedimentation in a driving syringe, the syringe pump is set to a high flow rate (*Q_sp_* = 10 mL/h), and blood behaves as a Newtonian fluid. Blood viscosity is then obtained by substituting time-lapse flow rates into an analytical expression derived from a fluidic circuit model. The overall blood delivery time is less than 200 s.

## 2. Materials and Methods

### 2.1. A Microfluidic Rheometry for Probing Biomechanical Properties from Blood Flows

**Figure 1:**
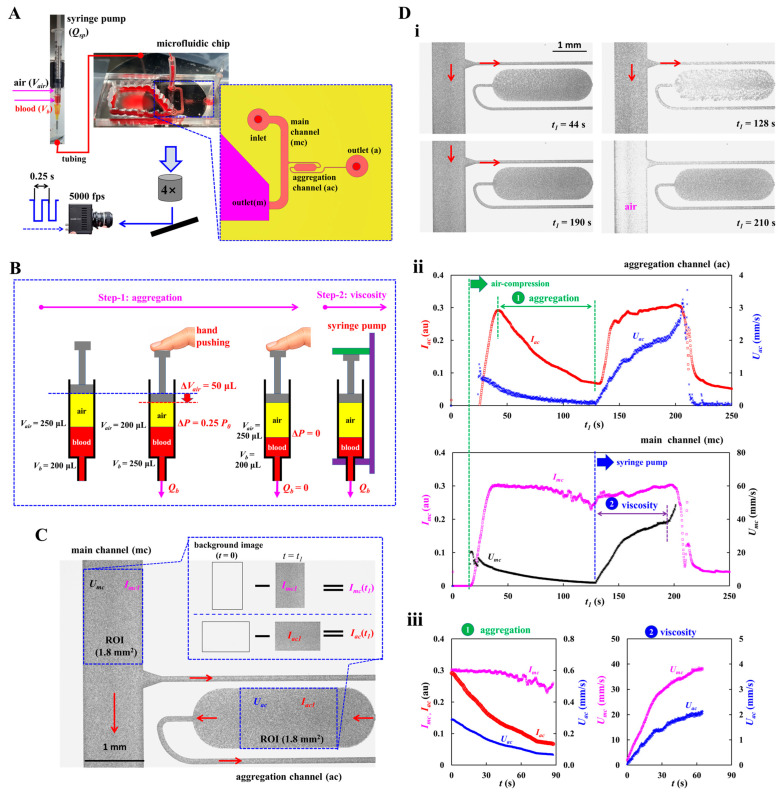
A proposed microfluidic rheometry for probing RBC aggregation and blood viscosity. (**A**) Experimental setup, including a microfluidic chip, a single syringe pump, and an imaging acquisition system. A microfluidic chip was designed to have an inlet, main channel (mc), aggregation channel (ac), and two outlets. A disposable syringe was partially filled with air (*V_air_* = 250 μL) and blood (*V_b_* = 100~200 μL), and connected to the inlet with a polyethylene tubing (i.d. = 0.25 mm, length = 300 mm). Blood flow images were recorded using an imaging acquisition setup consisting of a microscope (4× objective lens, NA = 0.1) and a high-speed camera operating at 5000 frames per second. An external trigger interval is set to a specific period (*T* = 0.25 s). (**B**) Two steps of blood delivery (i.e., manual air compression for RBC aggregation, and syringe pump for blood viscosity). (**C**) Quantification of blood velocity and blood imaging intensity in the microfluidic channels. Blood velocity (*U_mc_*) and imaging intensity (*I_mc_*) were evaluated by selecting a specific ROI (1.8 mm^2^) in the main channel. Similarly, blood velocity (*U_ac_*) and imaging intensity (*I_ac_*) were obtained from a specific ROI (1.8 mm^2^) positioned within the large-sized chamber of the aggregation channel. (**D**) Preliminary demonstration of the suggested method. Herein, test blood (hematocrit [Hct] = 50%) was prepared by suspending normal RBCs into dextran solution (20 mg/mL). Blood (*V_b_* = 200 μL) was loaded into a syringe. Blood flow rate set to *Q_sp_* = 10 mL/h. (**i**) Time-lapse blood flow imaging with an elapse of time (*t*_1_ = 44, 128, 190, and 210 s). The arrow indicated blood flow direction in the main channel and aggregation channel. (**ii**) Time-lapse image intensity (*I_mc_*, *I_ac_*) and blood velocity (*U_mc_*, *U_ac_*). Firstly, using manual delivery of a syringe, the compliance effect in the syringe contributed to transient blood flow. Due to RBC aggregation in the aggregation channel, the *I_ac_* was decreased over time substantially. Secondly, by supplying blood with a syringe pump, time-lapse *U_mc_* and *U_ac_* were used to obtain blood viscosity. (**iii**) Time-lapse data sets selected for assessing RBC aggregation and blood viscosity.

As shown in Figure 1A, to get flow-dependent RBC aggregation and blood viscosity, a suggested experimental setup was composed of a microfluidic chip, a blood delivery method, and an imaging acquisition system.

A microfluidic chip was designed to have an inlet, a main channel (mc), an aggregation channel (ac), and two outlets (m and a). As a key design concept, to minimize RBC sedimentation in a driving syringe, the flow rate is set to a high value of *Q_sp_* = 10 mL/h with a syringe pump. As a shear rate was estimated as $\dot{\gamma }>10^{3}$ s^−1^ in the main channel (width = 1 mm, and length = 14.6 mm), RBCs were fully disaggregated. Thus, it was certain that blood viscosity remained consistent with respect to the higher shear rates. On the other hand, to probe RBC aggregation, the aggregation channel was bifurcated from the main channel. Based on the fluidic resistance formula of a rectangular channel with low aspect ratio (i.e., *R_f_* = $\frac{12\;\mu \;L}{w\;h^{3}}$, *μ*: viscosity, *w*: width, *h*: depth, *L*: length) [42], the fluidic resistance of the aggregation channel increased substantially by decreasing channel width. That is, RBC aggregation did not occur in the narrow-width channel. RBC aggregation was generated by intentionally positioning a wide-width channel region between the narrow-width channels [15]. The aggregation channel was then designed to have three segments connected in series: the first channel (width = 0.1 mm, length = 4.9 mm), the second channel (width = 1 mm, length = 2 mm) and the third channel (width = 0.1 mm, length = 8.8 mm). All channels had the same channel depth of *h* = 0.05 mm.

A four-inch silicon master mold was produced using standard microelectromechanical system processes, including photolithography and deep reactive ion etching. PDMS (Sylgard 184, Dow Corning, Midland, MI, USA) was prepared by mixing the elastomer base and curing agent at 10:1 (*w*/*w*). To remove entrapped air, the mixture was degassed under vacuum for 1 h. The PDMS was then cured in a convection oven at 70 °C for 1 h, peeled off from the master, and trimmed with a razor blade. One inlet and two outlets (m and a) were formed using a biopsy punch (outer diameter = 2 mm). The PDMS block was subsequently bonded to a glass substrate via oxygen plasma treatment (CUTE-MPR, Femto Science Co., Hwaseong-si, Republic of Korea). To enhance adhesion between the PDMS channel walls and the glass substrate, the microfluidic chip was heated on a hot plate at 120 °C for 10 min [79].

To minimize non-specific adsorption of plasma proteins on the inner channel surfaces, 0.2% bovine serum albumin (BSA) solution was introduced into the microfluidic channels. After 10 min of incubation, the BSA solution was removed by flushing the device with 1× PBS.

An air cavity secured in a driving syringe was employed to minimize blood loss resulting from dead volume in a fluidic path. After attaching a 20-gauge needle to the syringe, air (*V_air_* = 250 μL) and blood (*V_b_* = 100~200 μL) were sequentially aspirated into the driving syringe. The air cavity was then positioned above the blood against the gravitational direction. A polyethylene tubing (i.d. = 0.25 mm, and length = 300 mm) was connected between the syringe needle tip and the inlet port.

As shown in Figure 1B, to effectively measure RBC aggregation and blood viscosity, blood was supplied with two different delivery methods (i.e., manual air-compression for RBC aggregation, and syringe pump for blood viscosity). First, air trapped along the fluidic pathway was expelled by compressing the air pocket in the driving syringe from 250 to 200 μL [79]. Based on an ideal-gas law (i.e., air pressure × air volume = constant) [42,50], air pressure increased to *P* = 1.25 *P*_0_. Herein, the *P*_0_ denotes atmospheric pressure (*P*_0_ = 101 kPa). Air pressure difference (Δ*P* = 0.25 *P*_0_) contributed to loading blood into a microfluidic chip from the syringe. After expelling air through the outlets, all channels became fully filled with blood. As the air cavity was increased gradually to the set value, the air pressure difference was also decreased gradually to zero. Accordingly, when the blood flow rate was reduced below a threshold, RBC aggregation occurred in the aggregation channel. In contrast, the blood flow rate in the main channel remained sufficiently high to keep RBCs fully disaggregated. By comparing the image intensity of blood in the two channels, RBC aggregation could be quantified. Second, after an elapse of a certain time (about 120 s), to measure blood viscosity, a syringe pump is set to a constant value of flow rate (*Q_sp_* = 10 mL/h). Aggregated RBCs were fully dispersed at the higher flow rate. Owing to the air-compliance effect, the blood flow rate rose progressively over time before stabilizing at a plateau value. Blood viscosity was acquired by analyzing time-lapse blood velocity in the main channel and aggregation channel.

The microfluidic device was placed on an inverted microscope (IX81, Olympus, Tokyo, Japan) equipped with a 4× objective (NA = 0.10). Bloodflow images were acquired using a high-speed camera at 5000 frames per second, with an external trigger interval set to *T* = 0.25 s. All experiments were conducted at a constant room temperature of 25 °C.

### 2.2. Quantification of Image Intensity and Blood Flow Rate in Main and Aggregation Channels

In this study, variation of RBC aggregation was quantified by comparing image intensities of blood flow in the main channel (i.e., fully disaggregated RBCs) and the aggregation channel (i.e., aggregated RBCs), respectively. In addition, to obtain blood viscosity, the time-dependent flow rate in the main channel and aggregation channel was required. For these reasons, it was necessary to obtain image intensity as well as blood velocity in both channels.

As shown in Figure 1C, two ROIs (regions of interest) were selected in the main channel and aggregation channels. The area of each ROI was set to 1.8 mm^2^. Bloodflow direction in the microfluidic channels was marked by red arrows. The scalar bar denoted 1 mm.

First, to assess the contribution of RBCs in each channel, a subtracted image was calculated by subtracting each image from the initial background [28]. Herein, the RBC contribution was used to quantify the magnitude of RBC aggregation. All calculations were performed using an image processing toolbox in MATLAB (Version: 2025b, MathWorks, Natick, MA, USA). Based on ROIs defined in the main and aggregation channels, the mean value of grayscale intensity was calculated as *I_mc_* (main channel) and *I_ac_* (aggregation channel), respectively. Time-lapse image intensity was subsequently determined by applying the same image-processing procedure to all recorded images.

Second, time-lapse velocity fields were measured using open source PIV software (PIVlab, version: 3.12) [80]. To obtain velocities within each ROI, an interrogation window of 13 × 13 µm^2^ with 50% overlap was used [15]. The resulting velocity vectors were post-processed using local median and standard-deviation filters. Based on the analytical depth-of-correlation (DOC) formula [81], the DOC of the imaging system (Figure 1A) was estimated as DOC > 300 µm. Because the DOC was much larger than the channel depth (i.e., DOC > *h*), the micro-PIV results were considered as depth-averaged velocity within each interrogation window. Mean velocity was calculated by averaging velocity values over each ROI, yielding *U_mc_* for the main channel and *U_ac_* for the aggregation channel. Time-lapse velocities were then obtained by repeating the same micro-PIV procedure for all recorded images. Considering that blood velocity had been strongly impacted by hematocrit [82], it was necessary to calibrate velocity fields obtained by the micro-PIV technique. To perform a simple calibration procedure, the syringe pump is set to a constant value of flow rate (*Q_sp_*). Owing to the air compliance effect in a driving syringe, blood velocity increased gradually and reached a steady value (*U_st_*) after a certain period. The blood flow rate in each channel was calculated from *Q_sp_* and *U_st_*, eliminating the need for any further calibration steps. That is, the corresponding flow rate in each channel was compensated as *Q_mc_*(*t*) = *U_mc_*(*t*)/*U_st_* × *Q_sp_* and *Q_ac_*(*t*) = *U_ac_*(*t*)/*U_st_* × *Q_sp_*, respectively.

To demonstrate time-dependent image intensity and blood velocity, test blood (hematocrit [Hct] = 50%) was prepared by suspending normal RBCs into dextran solution (20 mg/mL). By referring to the previous studies [15], the specific concentration of dextran solution was selected as a blood medium for maximizing RBC aggregation. Blood volume of *V_b_* = 200 μL was suctioned into the syringe. Blood flow rate set to *Q_sp_* = 10 mL/h. As shown in Figure 1D(i), the blood image was summarized with respect to time (*t*_1_ = 44, 128, 190, and 210 s). Herein, the red arrow indicated bloodflow direction in channels. The microscopic image acquired at *t*_1_ = 128 s showed clearly enhanced RBC aggregation compared with the image acquired at *t*_1_ = 44 s. Specifically, the ROI in the aggregation channel exhibited higher brightness than the corresponding region in the main channel. Finally, at *t*_1_ = 210 s, the syringe delivered air into the main channel, which resulted in stopping blood flows completely. However, owing to higher fluidic resistance, air did not invade the aggregation channel, which was filled only with blood.

As depicted in Figure 1D(ii), the suggested protocols were used to get time-dependent image intensity (*I_mc_*, *I_ac_*) and blood velocity (*U_mc_*, *U_ac_*). The upper panel showed temporal variations of *I_ac_* and *U_ac_* obtained in the aggregation channel. The lower panel depicted time-lapse *I_mc_* and *U_mc_* acquired in the main channel. Firstly, as shown in the green-dashed line, air volume (*V_air_* = 50 μL) in a deriving syringe was compulsorily compressed to load blood. Because the pressure difference between the air pressure in the syringe and atmospheric pressure increased, blood was supplied into the microfluidic channels through the fluidic path. As air volume increased over time, the pressure difference decreased over time. For this reason, blood velocity (*U_mc_*, *U_ac_*) tended to decrease over time. The *I_mc_* remained unchanged for up to *t*_1_ = 90 s. After *t*_1_ = 90 s, it tended to decrease slightly over time, which denoted that RBC aggregation occurred in the fluidic path. Aggregated RBCs came into the microfluidic channels. As the shear rate in the aggregation channel decreased sufficiently below a threshold ($\dot{\gamma }$ = 50~100 s^−1^), RBC aggregation occurred in the aggregation channel. Transiently decreasing blood flow contributed to increasing *I_ac_* significantly. Thus, the RBC aggregation index (AI) could be obtained continuously by analyzing time-dependent *I_mc_* and *I_ac_*, respectively. Secondly, as depicted in the blue-dashed line, the syringe pump set to a constant flow rate (*Q_sp_*). Due to the air-compliance effect in a driving syringe, *U_mc_* and *U_ac_* increased gradually and reached a plateau value. The *I_ac_* increased gradually because full disaggregated RBCs flowed into both channels. Above *t*_1_ = 199 s, the *I_mc_* tended to decrease. The *U_mc_* tended to increase significantly. As *U_mc_* and *U_ac_* were strongly impacted by blood viscosity, they participated in obtaining blood viscosity. As shown in Figure 1D(iii), time-lapse data sets were selected for assessing RBC aggregation and blood viscosity. To probe RBC aggregation, an initial time (*t* = 0) was reset at a specific time when the *I_ac_* had maximum value. The final time (*t* = *t_end_*) was then determined at the time when the *I_ac_* had a low saturated value. Time-lapse *I_mc_*, *I_ac_*, and *U_ac_* were plotted from *t* = 0 to *t* = *t_end_*. On the other hand, for assessing blood viscosity, an initial time began from the time when the syringe pump turned on. The final time was set at the time when *Q_mc_* arrived at the plateau value. Temporal variations of *U_mc_* and *U_ac_* were plotted from *t* = 0 to *t* = *t_end_*.

From the preliminary demonstration, image intensity (*I_mc_*, *I_ac_*) and blood velocity (*U_mc_*, *U_ac_*) could be used effectively to probe RBC aggregation and blood viscosity.

### 2.3. Mathematical Representation of Proposed Microfluidic System

**Figure 2:**
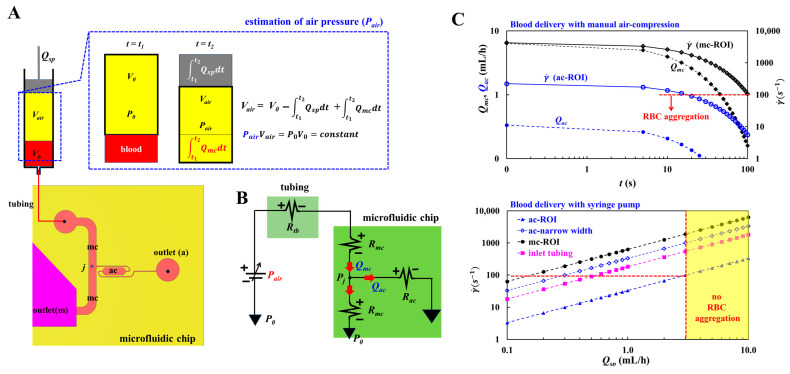
Mathematica representation of blood viscosity. (**A**) Estimation of air pressure in a driving syringe (*P_air_*). (**B**) Fluidic circuit model of the proposed microfluidic platform. The upper panel showed a discrete fluidic circuit model, including a pressure source (*P_air_*), fluidic resistance element (*R_tb_*: inlet tubing, *R_mc_*: main channel, and *R_ac_*: aggregation channel). The ground, *P*_0_, denoted an atmospheric pressure (*P*_0_ = 101 kPa). The *P_j_* denotes blood pressure at the junction between the main channel and the aggregation channel. Based on fluidic circuit analysis, pressure difference ($∆P=\;P_{air}-P_{0}$) was derived as $∆P=\left(R_{tb}+2R_{mc}\right){\;Q}_{mc}-R_{mc}\;Q_{ac}$. (**C**) Variations of shear rate ($\dot{\gamma }$) in fluidic path (i.e., inlet tubing, ROI in the main channel, and ROI in the aggregation channel) with respect to blood delivery (i.e., manual air compression and syringe pump). The upper panels exhibited time-lapse flow rate (*Q_mc_*, *Q_ac_*) and shear rates in the main channel and aggregation channel ($\dot{\gamma }$[ac-ROI], and $\dot{\gamma }$[mc-ROI]). The lower panels showed variations as a function of syringe flow rate (*Q_sp_*). Red-dash line denoted initialization of RBC aggregation ($\dot{\gamma }=100\;s^{-1})$.

To derive the blood viscosity formula, firstly, it was necessary to obtain the air pressure in the syringe. As shown in Figure 2A, a driving syringe partially filled with air and blood was positioned against the gravitational direction. A polyethylene tube was connected between the needle tip and the inlet port. Initially (*t* = *t*_1_), air volume was defined as *V_air_* = *V*_0_ and air pressure equaled atmospheric pressure (*P_air_* = *P*_0_). Over the period from *t* = *t*_1_ to *t* = *t*_2_, the air cavity decreased by $\int_{t_{1}}^{t_{2}}Q_{sp}dt$ because piston moved downward. In contrast, the air cavity increased by $\int_{the\;t_{1}}^{t_{2}}Q_{mc}dt$ because the volume decreased at the flow rate of *Q_mc_*. Based on the mass balance law in a driving syringe, air volume (*V_air_*) inside the syringe was estimated as,

(1) $$V_{air}\left(t_{2}\right)=V_{0}-\int_{t_{1}}^{t_{2}}Q_{sp}dt+\int_{t_{1}}^{t_{2}}Q_{mc}dt.$$

According to the ideal-gas law [50] (i.e., $P_{air}\times V_{air}=P_{0}\times V_{0}=constant)$, the air pressure (*P_air_*) inside the driving syringe was determined as *P_air_* = *P*_0_ × *V*_0_/*V_air_*. The analytical expression of *P_air_* was derived as,

(2) $$P_{air}\left(t_{2}\right)=\frac{P_{0}\times V_{0}}{V_{0}-\int_{t_{1}}^{t_{2}}Q_{sp}dt+\int_{t_{1}}^{t_{2}}Q_{mc}dt}.$$

Using Equation (2), the pressure difference (Δ*P* = *P_air_* − *P*_0_) was given as,

(3) $$\Delta P=P_{0}\left(\frac{V_{0}}{V_{0}-\int_{t_{1}}^{t_{2}}Q_{sp}dt+\int_{t_{1}}^{t_{2}}Q_{mc}dt}-1\right).$$

According to the Equation (3), the pressure difference (Δ*P*) could be obtained consistently if time-dependent *Q_mc_* was obtained accurately.

Next, a fluidic circuit model was constructed to derive an analytical expression of blood viscosity. As shown in Figure 2B, the fluidic circuit model of the proposed microfluidic platform was composed of an air pressure source (*P_air_*), fluidic resistance element (i.e., *R_tb_*: inlet tubing, *R_mc_*: main channel, and *R_ac_*: aggregation channel). Herein, assuming that blood behaved as a Newtonian fluid, the ratio of *R_mc_* to *R_ac_* was calculated as *R_mc_*/*R_ac_* = 19.04. Accordingly, the flow was split such that 5% of the supplied blood passed through the aggregation channel, whereas the other 95% proceeded through the main channel. The ground (‘▼’) denoted atmospheric pressure (*P*_0_ = 101 kPa). The *P_j_* denotes blood pressure at the junction between the main channel and the aggregation channel. With regard to fluidic path (i.e., syringe–inlet tubing–upper main channel–junction point), pressure difference (i.e., *P_air_* − *P_j_*) was derived as,

(4) $$P_{air}-P_{j}=\left(R_{tb}+R_{mc}\right)Q_{mc}.$$

In addition, with regard to lower main channel (i.e., junction point–lower main channel–outlet [m]), pressure difference (i.e., *P_j_* − *P*_0_) was derived as,

(5) $$P_{j}-P_{0}=R_{mc}(Q_{mc}\;-Q_{ac}).$$

By summing Equations (4) and (5), pressure difference (Δ*P* = *P_air_* − *P*_0_) was given as,

(6) $$\Delta P=\left(R_{tb}+{2R}_{mc}\right)Q_{mc}\;-R_{mc}Q_{ac}.$$

In Equation (6), the formula of *R_mc_* and *R_tb_* were analytically given as,

(7) $$R_{mc}=\frac{12\;\mu_{b}L_{mc}}{w\;h^{3}},$$

(8) $$\mathrm{and}\;R_{tb}=\frac{8\;\mu_{b}L_{tb}}{\pi \;r^{4}}.$$

In Equations (7) and (8), *L_mc_* and *L_tb_* denoted channel length of the main channel and inlet tubing. The *r* meant the inner radius of the inlet tubing. The pressure difference was then simplified as

(9) $$\Delta P=\mu_{b}({\epsilon \;Q}_{mc}\;-\lambda {\;Q}_{ac}).$$

In Equation (9), the ϵ and *λ* were given as

(10) $$\epsilon =\frac{12\;L_{mc}}{w\;h^{3}}\;+\frac{8\;L_{tb}}{\pi \;r^{4}},$$

(11) $$\mathrm{and}\;\lambda =\frac{6\;L_{mc}}{w\;h^{3}}.$$

Using Equation (9), the analytical formula of blood viscosity was finally derived as,

(12) $$\mu_{b}=\frac{\Delta P}{\left({\epsilon \;Q}_{mc}-\lambda \;Q_{ac}\right)}.$$

In the Equation (12), considering that ϵ and *λ* were fixed, blood viscosity (*μ_b_*) could be then obtained from time-lapse *Q_mc_*, *Q_ac_*, and Δ*P*.

At last, Figure 2C exhibited variations of shear rate ($\dot{\gamma }$) in fluidic path (i.e., inlet tubing, ROI in the main channel, and ROI in the aggregation channel) with respect to blood delivery (i.e., manual air-compression for RBC aggregation, and syringe pump for blood viscosity). The upper panels exhibited time-lapse flow rate (*Q_mc_*, *Q_ac_*) and shear rates in the main channel and aggregation channel ($\dot{\gamma }$[ac-ROI], $\dot{\gamma }$[mc-ROI]). As shown in Figure 2B, the flow rate in each channel (*Q_mc_*, *Q_ac_*) was estimated by analyzing a fluidic circuit model. Shear rate was then calculated by substituting flow rate and dimensional values into the shear-rate expression (i.e., $\dot{\gamma }=\frac{6Q}{w\;h^{2}}$, *Q*: flow rate in each channel) [42]. Red-dash line denoted threshold value of RBC aggregation ($\dot{\gamma }=100\;s^{-1})$. Within the ROI of the aggregation channel, the estimated shear rate was below the threshold ($\dot{\gamma }$ = 100 s^−1^). In contrast, the shear rate in the main channel was estimated to exceed the threshold. That is, RBC aggregation could be observed when blood flows within the ROI of the aggregation channel, whereas RBC aggregation could not be detected in the main channel. By comparing image intensity in each channel, it is possible to obtain the RBC aggregation index under continuous blood flow. The lower panels exhibited variations of shear rate with respect to *Q_sp_*. Herein, the *Q_sp_* denoted constant flow rate of the syringe pump. The *Q_sp_* ranged from 0.1 mL/h to 10 mL/h. Based on the shear rate formula of a rectangular channel and a circular tubing ($\dot{\gamma }=\frac{4Q}{\pi \;r^{3}}$, *Q*: blood flow rate) [42], the corresponding shear rate of each channel was simulated with respect to *Q_sp_*. From the simulation results, below *Q_sp_* = 1 mL/h, RBC aggregation occurred in the aggregation channel. Above *Q_sp_* = 3 mL/h, shear rate was estimated as above $\dot{\gamma }$ = 100 s^−1^ for all channels. As aggregated RBCs were fully disaggregated, their contribution to blood viscosity could be neglected under the blood delivery of the syringe pump.

### 2.4. Preparation of Test Blood

Concentrated red blood cells were supplied by the Gwangju–Chonnam Blood Bank (Gwangju, Republic of Korea) and stored under refrigerated conditions prior to experimental preparation. Following established washing procedures [83], normal RBCs were isolated by sequentially removing blood-suspended medium and the buffy coat.

First, to visualize velocity fields of glycerin solution, normal RBCs (30 μL) were added to 1 mL of each concentration of glycerin solution. Second, to assess the effect of hematocrit on RBC aggregation and blood viscosity, the hematocrit of the test blood was adjusted to Hct = 30~50% by suspending normal RBCs into dextran solution (20 mg/mL). Third, to examine how the suspending medium influences these hemorheological properties, normal RBCs were suspended in dextran solution (*C_dex_* = 5~20 mg/mL), which was prepared by dissolving dextran powder (*Leuconostoc* spp., MW 450–650 kDa; Sigma-Aldrich, St. Louis, MO, USA) into 1× PBS. Herein, hematocrit was fixed at 50%. Finally, to investigate thermal-shocked effects, control blood (Hct = 50%) was prepared by suspending normal RBCs in 1× PBS. Using a thermomixer (Eppendorf, Hamburg, Germany), the control blood was incubated under heat-shock conditions (45 °C for up to 40 min or 50 °C for up to 20 min). Following the established washing protocols, test blood (Hct = 50%) was then prepared by suspending the thermally shocked RBCs in dextran solution (20 mg/mL).

### 2.5. Statistical Analysis

All statistical computations were carried out with MINITAB software (Version 22.4, Minitab Inc., State College, PA, USA). Under the assumption of normal distributed data, results were presented as mean ($\bar{x}$) ± standard deviation (σ), where *S_n_* represented experimental replication number. The bounds of 95% CI (confidence interval) were computed as $\bar{x}\;-1.96\frac{\;\sigma }{\sqrt{S_{n}}}$ and $\bar{x}\;+\;1.96\frac{\;\sigma }{\sqrt{S_{n}}}$. Statistical differences among groups were evaluated by one-way ANOVA. Statistical significance was set to *p*-value < 0.05 (95% CI).

## 3. Results and Discussion

### 3.1. Proposed Protocols of Flow-Dependent RBC Aggregation and Blood Viscosity

In this subsection, using time-lapse image intensity and blood velocity as illustrated in Figure 1D, the full methodology for quantifying flow-dependent RBC aggregation and blood viscosity was described in detail.

**Figure A1:**
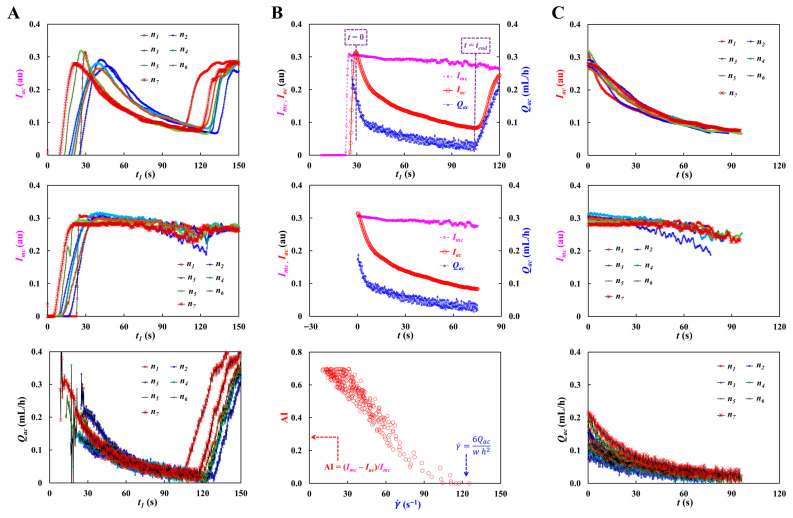
Test-to-test variability in terms of image intensity (*I_mc_*, *I_ac_*) and flow rate (*Q_ac_*) for quantifying consistent RBC aggregation index (AI). (**A**) Variations of image intensities and flow rate in the aggregation channel obtained from multiple reproductivity tests. (**B**) Resetting initial time (*t* = 0) for quantifying RBC aggregation with respect to shear rate. The first panel showed the selection of the initial time (*t* = 0) and final time (*t* = *t_end_*). Herein, the time at which the *I_ac_* reached its maximum was reset to the initial time (*t* = 0). The end time was also defined as the moment when the Iac reached its minimum value (*t* = *t_end_*). The second panel showed variations of *I_mc_*, *I_ac_*, and *Q_ac_* ranging from *t* = 0 to *t* = *t_end_*. Based on each formula of AI and shear rate, AI was then obtained as a function of shear rate. The last panel exhibited variations of AI with respect to shear rate. (**C**) Time-lapse image intensity and flow rate for quantifying RBC aggregation. Based on data selection procedures, the panels exhibited temporal variations of *I_mc_*, *I_ac_*, and *Q_ac_*.

As shown in Figure A1A (Appendix A), variations of multiple reproductivity were monitored in terms of image intensity (*I_mc_*, *I_ac_*) and flow rate (*Q_ac_*). Manual air compression was applied to load blood into a microfluidic channel, resulting in a transiently decreasing flow rate. Due to test-to-test variability, time-lapse traces of *I_mc_*, *I_ac_*, and *Q_ac_* showed different profiles. Based on the formula of RBC aggregation index (i.e., AI = Δ*I*/*I_mc_*, Δ*I* = *I_mc_* − *I_ac_*), as shown in Figure A1B (Appendix A), data were selected beginning at the initial time corresponding to the minimum AI value, and the final time was fixed at the point when AI reached its maximum plateau level. Based on the bounded time (i.e., initial time and final time), time-lapse *I_mc_*, *I_ac_*, and *Q_ac_* were replotted from *t* = 0 to *t* = *t_end_*. Based on the formula of RBC aggregation index and shear rate, the AI was plotted as a function of shear rate.

Following data-selection procedures, the time-lapse *I_mc_*, *I_ac_*, and *Q_ac_* signals extracted from the defined interval (Figure A1C, Appendix A) were used to assess RBC aggregation index as a function of shear rate.

**Figure 3:**
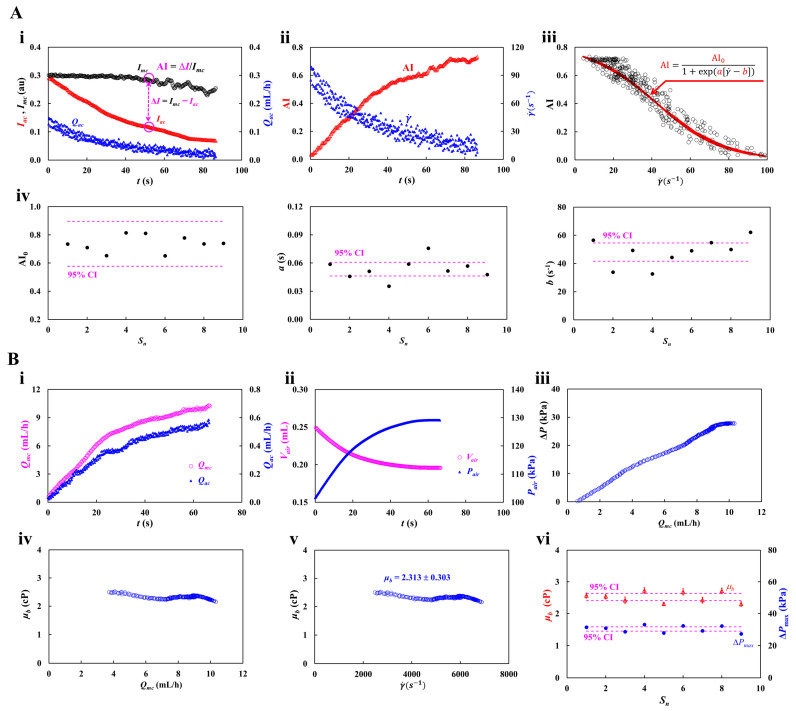
Quantification procedures of flow-dependent RBC aggregation and blood viscosity. (**A**) Assessment of shear-lapse RBC aggregation. (**i**) Temporal variations of *I_ac_*, *I_mc_*, and *U_ac_*. At a lower flow rate of the aggregation channel, RBC aggregation caused to decrease in *I_ac_* over time gradually. RBC aggregation index (AI) as dimensionless parameter was expressed as AI = Δ*I*/*I_mc_*, where the Δ*I* was defined as Δ*I* = *I_mc_* − *I_ac_*. (**ii**) Temporal variations of AI and shear rate ($\dot{\gamma }$). (**iii**) Variations of AI with respect to shear rate ($\dot{\gamma }$). To assess AI quantitatively, the AI was best fitted as AI = $\frac{{AI}_{0}}{1+exp(a\left[\dot{\gamma }-b\right])}$. Non-linear regression analysis gave three unknown parameters (i.e., *AI*_0_ = 0.8113, *a* = 0.059 s, and *b* = 44.3671 s^−1^). (**iv**) Variations of three parameters obtained for nine bloods (*S_n_* = 9). The dashed lines indicate the bounds of 95% confidence interval (CI). Based on the 95% CI, three parameters were obtained as 0.577 < *AI*_0_ < 0.896, 0.0463 s < *a* < 0.0608 s, and 41.641 s^−1^ < *b* < 54.506 s^−1^. (**B**) Assessment of flow-dependent blood viscosity. (**i**) Time-lapse *Q_mc_* and *Q_ac_*. (**ii**) Temporal variations of *V_air_* and *P_air_*. (**iii**) Variations of Δ*P* with respect to *Q_mc_*. (**iv**) Variations of blood viscosity *(μ_b_*) with respect to *Q_mc_*. (**v**) Variations of *μ_b_* with respect to $\dot{\gamma }$. The *μ_b_* remained constant with respect to shear rate. (**vi**) Variations of *μ_b_* and Δ*P_max_* obtained for nine bloods (*S_n_* = 9). Based on the 95% CI, the *μ_b_* and Δ*P_max_* were obtained as 2.416 cP < *μ_b_* < 2.629 cP and 28.981 kPa < Δ*P_max_* < 31. 732 kPa.

As shown in Figure 3A(i), shear-dependent RBC aggregation was quantified using time-lapse *I_mc_*, *I_ac_*, and *Q_ac_*. Initially, due to the air-compliance effect in a driving syringe, blood was loaded into a microfluidic chip from the syringe. With the elapse of the period, the air cavity inside the syringe increased over time. As air pressure decreased over time, the blood flow rate in the aggregation channel (*Q_ac_*) tended to decrease gradually over time. The *I_mc_* remained relatively constant for a certain time (*t* = 40 s). Above *t* = 40 s, it tended to decrease slightly over time. That is, as the delivery flow rate decreased sufficiently, RBC aggregation occurred from the syringe needle to the inlet port. The aggregated RBCs were flowed into the main channel and aggregation channel. Thus, the *I_ac_* decreased significantly over time.

From the experimental investigation, image intensity difference (Δ*I* = *I_mc_* − *I_ac_*) was strongly related to RBC aggregation [15]. To make the RBC aggregation dimensionless parameter, the Δ*I* was normalized by *I_mc_*. Thus, the RBC aggregation index (AI) was defined as AI = Δ*I*/*I_mc_*. The flow-dependent AI could be evaluated from time-lapse *I_ac_* and *I_mc_*. A novel RBC aggregation index adopted by the present method was already compared quantitatively with the conventional RBC aggregation index. The conventional index was calculated from temporal variations of image intensity after blood flow was stopped. However, our suggested index was calculated by analyzing the blood image in continuous blood flow. According to a quantitative comparison study, the suggested method gave consistent results obtained by the conventional index [15]. Thus, the newly suggested index was adopted to probe variations of RBC aggregation. Based on time-lapse *Q_ac_*, as shown in Figure 3A(ii), variations of AI and $\dot{\gamma }$ were obtained over time. Herein, based on the shear rate formula of a rectangular channel (i.e., $\dot{\gamma }=\frac{6\;Q_{ac}}{w\;h^{2}}$, *w* = 1 mm, *h* = 0.05 mm) [42], the shear rate within the ROI of the aggregation channel was estimated over time. Initially, the AI was estimated as near zero, where shear rate was estimated as about $\dot{\gamma }$ = 90 s^−1^. Considering that the previous studies [60] reported a threshold for RBC aggregation as $\dot{\gamma }$ = 50~100 s^−1^, the initial value of AI was regarded as reasonable. Furthermore, when the shear rate decreased gradually over time, the AI increased significantly.

To analyze shear-dependent AI quantitatively, as shown in Figure 3A(iii), variations of AI were represented with respect to $\dot{\gamma }$. According to the previous study [84], variations of AI were best fitted using a sigmoidal function and a power-law function. Considering that AI had steady value at low shear rates, for convenience, the sigmoidal function was selected to fit shear-dependent AI. That is, to probe shear-dependent AI quantitatively, the AI was best fitted as AI = $\frac{{AI}_{0}}{1+exp(a\left[\dot{\gamma }-b\right])}$. Based on a curve-fitter toolbox in MATLAB (Version: 2025b, MathWorks, Natick, MA, USA), non-linear regression analysis was carried out for estimating three unknown parameters (i.e., *AI*_0_, *a*, and *b*). The red line indicated the best-fit function, which accurately described the shear-dependent AI. Three unknown parameters were then extracted as *AI*_0_ = 0.8113, *a* = 0.059 s, and *b* = 44.3671 s^−1^. The regression coefficient was obtained as a high value of R^2^ = 0.9359. Reproducibility of the proposed method was assessed by measuring variations in three parameters across nine test bloods (*S_n_* = 9). Figure 3A(iv) exhibited variations of three parameters (i.e., *AI*_0_, *a*, and *b*) with respect to test blood. The dashed lines indicate both bounds of 95% CI. Specifically, within the 95% CI, three parameters were estimated as 0.577 < *AI*_0_ < 0.896, 0.0463 s < *a* < 0.0608 s, and 41.641s^−1^ < *b* < 54.506 s^−1^.

**Figure A2:**
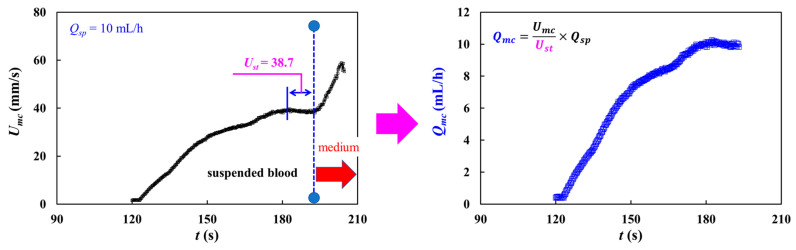
Calibration of *Q_mc_* using the average velocity over the plateau interval. Blood velocity in the main channel (*U_mc_*) increased gradually and reached a plateau. Based on a vertical blue-color line, blood medium (i.e., RBC-depleted blood) began to flow in the main channel because of RBC sedimentation in a driving syringe. It then increased sharply once the blood medium entered the main channel. Herein, the *U_st_* (steady state value) was defined as the average velocity over the plateau interval. At the flow rate of *Q_sp_* = 10 mL/h, the *U_st_* was obtained as *U_st_* = 38.7 mm/s. Blood flow rate in the main channel was calibrated as *Q_mc_* = *U_mc_*/*U_st_* × *Q_sp_*.

Next, using a syringe pump, blood was loaded into microfluidic channels. Blood viscosity could be estimated by analyzing time-lapse *Q_mc_* and *Q_ac_*. Within 95% confidential interval (*n* = 9), a steady value of blood velocity in the main channel was obtained as 38.57 mm/s < *U_st_* < 39.95 mm/s. Each flow rate was calibrated using a steady value of blood velocity in the main channel. As illustrated in Figure 1D(iii), the initial time (*t* = 0) and final time (*t* = *t_end_*) were identified from the time-lapse *U_mc_* data. The initial time corresponded to the moment when *U_mc_* began to increase after turning on a syringe pump. The end time marked the point at which *U_mc_* stabilized at its plateau level. As shown in Figure A2 (Appendix A), at the flow rate of *Q_sp_* = 10 mL/h, the *U_st_* was obtained as *U_st_* = 38.7 mm/s. Blood flow rate in the main channel was calibrated as *Q_mc_* = *U_mc_*/*U_st_* × *Q_sp_*. As shown in Figure 3B(i), due to the air-compliance effect in the driving syringe, *Q_mc_* and *Q_ac_* were increased gradually over time. Based on Equations (1) and (2), *V_air_* and *P_air_* were obtained over time. The air volume (*V_air_*) gradually decreased from 0.25 mL to 0.196 mL, while the air pressure (*P_air_*) increased over time and then stabilized at 129.15 kPa. Figure 3B(iii) depicted the variation of Δ*P* as a function of *Q_mc_*, where ΔP was defined as *P_air_* − *P*_0_. The maximum value of ΔP was given as Δ*P_max_* = 28.15 kPa. The Δ*P* was approximately proportional to *Q_mc_*. The blood viscosity (*μ_b_*) was then calculated by substituting time-resolved *Q_mc_*, *Q_ac_*, and Δ*P* into Equation (12). Figure 3B(iv) showed variations of blood viscosity *(μ_b_*) as a function of *Q_mc_*. The results indicated that blood viscosity remained consistent with respect to *Q_mc_*. Based on the shear-rate formula, time-lapse *Q_mc_* was converted into shear rate. As shown in Figure 3B(v), variations of *μ_b_* were then represented with respect to $\dot{\gamma }$. Above $\dot{\gamma }$ = 2500 s^−1^, the blood viscosity (*μ_b_*) remained constant with respect to $\dot{\gamma }$. As expected, blood behaved as a Newtonian fluid [85,86,87,88]. The viscosity was summarized as *μ_b_* = 2.313 ± 0.303 cP (*n* = 227). COV (coefficient of variance, standard deviation/mean) was calculated as 13.1%. With regard to nine test bloods (*S_n_* = 9), reproducibility of the proposed method was evaluated by measuring *μ_b_* and Δ*P_max_*. Under 95% CI, the *μ_b_* and Δ*P_max_* were estimated as 2.416 cP < *μ_b_* < 2.629 cP and 28.981 kPa < Δ*P_max_* < 31. 732 kPa.

From the preliminary demonstration, it was confirmed that the proposed method was able to measure flow-resolved AI and viscosity by analyzing time-lapse image intensity (*I_mc_*, *I_ac_*) and flow rate (*Q_mc_*, *Q_ac_*). The method gave consistent results sufficiently.

### 3.2. Accuracy Validation of Viscosity Measured by the Proposed Method for RBC-Free Solution

Given that blood viscosity is strongly influenced by several factors (i.e., hematocrit, RBC aggregation, RBC deformability, and RBC sedimentation in the driving syringe) [44,57,58,61,89,90], the presence of RBCs complicates reliable viscosity measurement. Accordingly, validation can be simplified by eliminating RBCs from the blood suspension [91,92,93]. The remaining medium behaves as a Newtonian fluid. In this subsection, to verify the accuracy of viscosity values obtained with the proposed method, glycerin solutions were prepared as test fluids. To demonstrate that the proposed method provided shear-rate independent viscosity, the viscosity of glycerin solution was evaluated by varying flow rate, ranging from *Q_sp_* = 1 mL/h to *Q_sp_* = 8 mL/h, using a syringe pump. In addition, to substantially elevate viscosity, glycerin concentration was increased from 20% to 50%. The measurement results were quantitatively compared with reference data [94].

**Figure 4:**
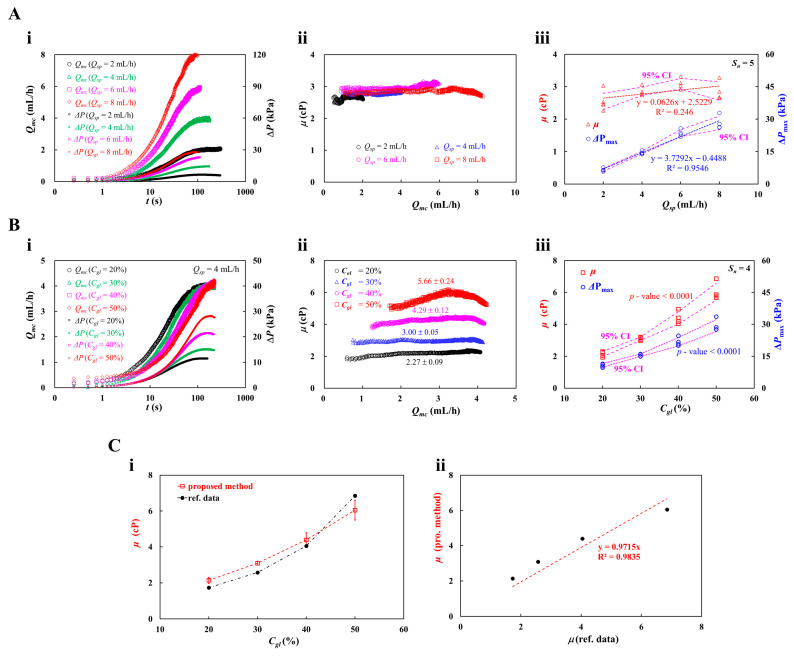
Accuracy validation of viscosity for glycerin solution. (**A**) Contribution of flow rate to viscosity. Herein, 30% glycerin solution was selected as the test fluid. (**i**) Time-lapse *Q_mc_* and Δ*P* with respect to *Q_sp_* = 2, 4, 6, and 8 mL/h. (**ii**) Variation of viscosity (*μ*) with respect to *Q_mc_* and *Q_sp_*. The viscosity was independent of *Q_mc_* and remained unchanged. (**iii**) Variations of *μ* and Δ*P_max_* with respect to *Q_sp_*. (**B**) Contribution of glycerin concentration (*C_gl_*) to viscosity. Herein, the flow rate was fixed at *Q_sp_* = 4 mL/h. (**i**) Time-lapse *Q_mc_* and Δ*P_max_* with respect to concentration of glycerin solution (*C_gl_* = 20%, 30%, 40%, and 50%). (**ii**) Variation of *μ* with respect to *Q_mc_* and *C_gl_*. (**iii**) Variations of *μ* and Δ*P_max_* with respect to *C_gl_*. (**C**) Quantitative comparison between the proposed method and reference data. (**i**) Variations of viscosity obtained by both methods with respect to *C_gl_*. (**ii**) Linear regression of viscosity obtained by both methods.

First, to validate Newtonian behavior of glycerin solution, as shown in Figure 4A(i), time-resolved *Q_mc_* and Δ*P* were summarized with respect to *Q_sp_* = 2, 4, 6, and 8 mL/h. Herein, 30% glycerin solution was selected as the test fluid. As expected, *Q_mc_* and Δ*P* increased gradually over time and eventually stabilized. The time required to reach the plateau decreased markedly at higher flow rates. Viscosity of the glycerin solution was estimated by substituting *Q_mc_*, *Q_ac_*, and Δ*P* into Equation (12). As shown in Figure 4A(ii), by adjusting *Q_sp_* ranging from 2 mL/h to 8 mL/h, the viscosity (*μ*) was plotted as a function of *Q_mc_*. From the results, the measured viscosity remained steady and did not correlate with *Q_mc_*. As expected, the glycerin solution behaved as a Newtonian fluid. The corresponding viscosity of each setting flow rate (*Q_sp_*) was estimated as *μ* = 2.66 ± 0.05 cP (*n* = 426) for *Q_sp_* = 2 mL/h, *μ* = 2.84 ± 0.05 cP (*n* = 668) for *Q_sp_* = 4 mL/h, *μ* = 3.03 ± 0.07 cP (*n* = 421) for *Q_sp_* = 6 mL/h, and *μ* = 2.84 ± 0.05 cP (*n* = 379) for *Q_sp_* = 8 mL/h. As depicted in Figure 4A(iii), variations of *μ* and Δ*P_max_* were plotted as a function of *Q_sp_*. For confirming reproducibility, the experiments were repeated five times (*S_n_* = 5). Linear regression analysis was carried out to find out the contributions of *Q_sp_* to *μ* and Δ*P_max_*. According to linear regression analysis, the regression coefficient of *μ_b_* gave a lower value of R^2^ = 0.246. The results indicated that the *μ_b_* did not show a substantial difference with respect to *Q_sp_*. The experimental results confirmed that the glycerin solution behaved Newtonian fluid. In addition, a strong regression fit was obtained for Δ*P_max_* (R^2^ = 0.9546), which confirmed that Δ*P_max_* was linearly proportional to *Q_sp_*. The results were consistent and reasonable because pressure was directly proportional to the delivered flow rate.

Second, to measure the accuracy of viscosity obtained by the proposed method, four different concentrations of glycerin solution (*C_gl_* = 20%, 30%, 40%, and 50%) were prepared by diluting pure glycerin solution with 1× PBS. Herein, the flow rate was fixed at *Q_sp_* = 4 mL/h. According to the reference data [94], the corresponding viscosity of each glycerin solution was given as *μ* = 1.72 cP for *C_gl_* = 20%, *μ* = 2.57 cP for *C_gl_* = 30%, *μ* = 4.05 cP for *C_gl_* = 40%, and *μ* = 6.86 cP for *C_gl_* = 50%. As shown in Figure 4B(i), time-lapse *Q_mc_* and Δ*P* were measured with respect to the concentration of glycerin solution (*C_gl_* = 20~50%). From the results, the *Q_mc_* was increased more slowly as the glycerin concentration increased. In contrast, the plateau value of Δ*P* increased substantially at higher concentrations of glycerin solution. As shown in Figure 4B(ii), by varying *C_gl_* ranging from 20% to 50%, the viscosity (*μ*) was plotted as a function of *Q_mc_*. Except higher concentration of *C_gl_* = 50%, the *μ* remained consistent with respect to *Q_mc_*. The corresponding viscosity of each concentration of glycerin solution was evaluated as *μ* = 2.27 ± 0.09 cP (*n* = 626) for *C_gl_* = 20%, *μ* = 3.00 ± 0.05 cP (*n* = 865) for *C_gl_* = 30%, *μ* = 4.29 ± 0.12 cP (*n* = 799) for *C_gl_* = 40%, and *μ* = 5.66 ± 0.24 cP (*n* = 783) for *C_gl_* = 50%. Figure 4B(iii) depicts variations of *μ* and Δ*P_max_* with respect to *C_gl_*. Experiments were repeated four times (*S_n_* = 4). The results showed that the CI width increased with higher glycerin concentration. To validate the performance of the proposed method, as shown in Figure 4C(i), viscosity values obtained by both methods (i.e., the proposed method and reference data) were plotted simultaneously with respect to *C_gl_*. From the results, both methods gave consistent results. To find out the linear correlation between both methods, as shown in Figure 4C(ii), viscosity values obtained by both methods were overlapped in a scatter plot, where the horizontal axis corresponded to the reference data (ref. data) and the vertical axis corresponded to the viscosity obtained by the proposed method (pro. method). The red dashed line indicated linear regression curve (i.e., *μ* [pro. method] = 0.9715 × *μ* [ref. data], R^2^ = 0.9835). Because the linear regression yielded a high value of the regression coefficient, the viscosity values obtained with the proposed method showed strong agreement with the reference data.

From the experimental investigations, the viscosity of glycerin solution remained constant with respect to the delivered flow rate. Viscosity exhibited a substantial difference with respect to the concentration of the glycerin solution. The quantitative evaluation confirmed that the proposed method could provide accurate viscosity.

### 3.3. Determination of Supplied Blood Flow Rate (Q_sp_) with Syringe Pump

RBC sedimentation in the driving syringe altered the hematocrit of the test blood during blood delivery [89,95,96,97]. In particular, hematocrit markedly influenced the blood velocity measured using time-resolved micro-PIV [98]. In this subsection, during syringe pump operation, selecting a suitable flow rate was essential to reduce the contribution of RBC sedimentation in the syringe. To intentionally enhance sedimentation in the syringe, a dextran solution of 20 mg/mL was chosen as the blood medium [15,65]. After performing RBC aggregation protocols for 120 s, the syringe pump was activated to load the blood into the microfluidic chip. For simplicity, blood viscosity was quantified only as a function of the applied flow rate. Herein, test blood (Hct = 50%) was prepared by mixing normal RBCs into 1× PBS and dextran solution (20 mg/mL). Each blood (*V_b_* = 200 μL) was loaded into the syringe. Flow rate of syringe pump set to *Q_sp_* = 2~10 mL/h.

**Figure 5:**
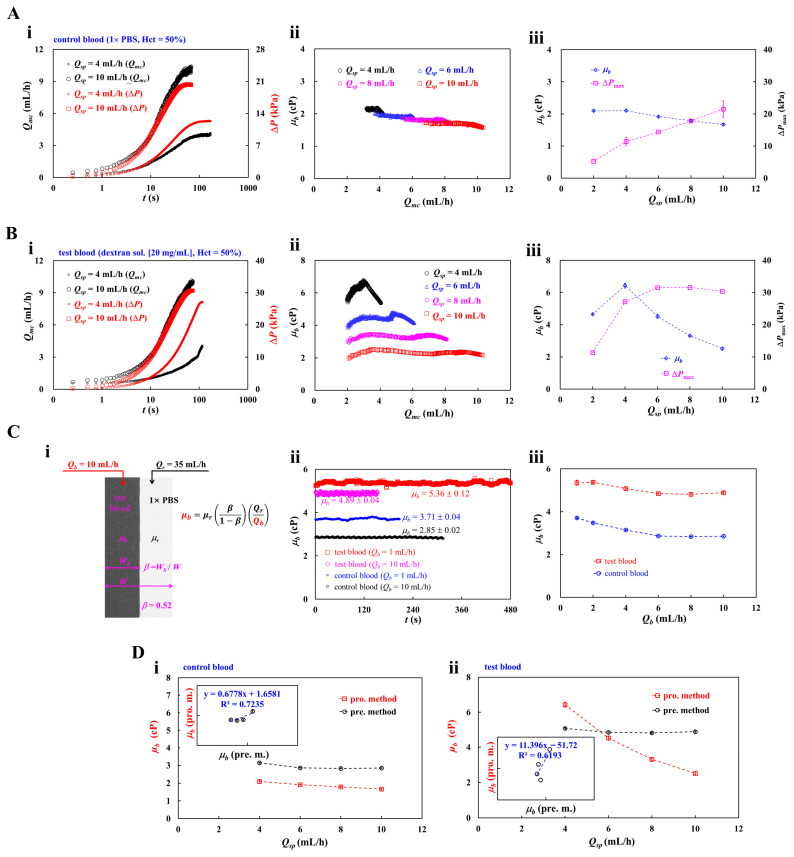
Determination of delivered blood flow rate for effectively measuring blood viscosity under RBCs sedimentation a driving syringe. Herein, two kinds of blood (Hct = 50%) were prepared by suspending normal RBCs in each blood medium (i.e., 1× PBS: control blood, and dextran solution [20 mg/mL]: test blood). (**A**) Viscosity of control blood obtained by the proposed method. (**i**) Time-lapse *Q_mc_* and Δ*P* with respect to *Q_sp_* = 4 and 10 mL/h. (**ii**) Variations of blood viscosity (*μ_b_*) with respect to *Q_mc_* and *Q_sp_* = 4, 6, 8, and 10 mL/h. (**iii**) Variations of *μ_b_* and Δ*P_max_* with respect to *Q_sp_*. (**B**) Viscosity of test blood obtained by the proposed method. (**i**) Time-lapse *Q_mc_* and Δ*P* with respect to *Q_sp_* = 4 and 10 mL/h. (**ii**) Variations of *μ_b_* with respect to *Q_mc_* and *Q_sp_*. (**iii**) Variations of *μ_b_* and Δ*P_max_* with respect to *Q_sp_*. (**C**) Viscosity of both bloods obtained by the previous method (i.e., coflowing streams method). (**i**) Blood viscosity assessment using the coflowing method. (**ii**) Temporal variations of *μ_b_* with respect to each blood and blood flow rate (*Q_b_* = 1 and 10 mL/h). (**iii**) Variations of *μ_b_* with respect to blood flow rate and each blood. (**D**) Quantitative comparison of blood viscosity obtained by the proposed method (pro. m.) and the previous method (pre. m.). (**i**) Quantitative comparison of *μ_b_* obtained by both methods for control blood. (**ii**) Quantitative comparison of *μ_b_* obtained by both methods for test blood.

First, to avoid RBC sedimentation in a driving syringe, 1× PBS was selected as the blood medium. Control blood (Hct = 50%) was prepared by suspending normal RBCs in 1× PBS. As shown in Figure 5A(i), time-lapse *Q_mc_* and Δ*P* were represented with respect to *Q_sp_* = 4 and 10 mL/h. Delivery time decreased as the delivered flow rate increased, whereas Δ*P* increased with increasing delivered flow rate. Based on time-lapse *Q_mc_* and Δ*P*, blood viscosity was obtained with respect to *Q_sp_*. As shown in Figure 5A(ii), by varying *Q_sp_* ranging from 4 mL/h to 10 mL/h, blood viscosity (*μ_b_*) was calculated and plotted as a function of *Q_mc_*. From the results, the variation ranges of *Q_mc_* were determined by the *Q_sp_*. Herein, within short variations of the *Q_mc_*, blood viscosity decreased slightly with respect to *Q_mc_*. For convenience, with respect to *Q_sp_*, blood viscosity was summarized as mean ± standard deviation (*n*: number of data points). That is, the corresponding viscosity of each *Q_sp_* was summarized as *μ* = 2.10 ± 0.05 cP (*n* = 371) for *Q_sp_* = 4 mL/h, *μ* = 1.92 ± 0.03 cP (*n* = 371) for *Q_sp_* = 6 mL/h, *μ* = 1.79 ± 0.03 cP (*n* = 311) for *Q_sp_* = 8 mL/h, and *μ* = 1.67 ± 0.04 cP (*n* = 191) for *Q_sp_* = 10 mL/h. As shown in Figure 5A(iii), blood viscosity (*μ_b_*) and maximum pressure difference (Δ*P_max_*) were plotted as a function of *Q_sp_* = 2~10 mL/h. From the results, above *Q_sp_* = 4 mL/h, overall variations of *μ_b_* tended to decrease gradually over *Q_mc_*. The results confirmed that control blood behaved as a non-Newtonian fluid. Furthermore, the delivered flow rate (*Q_sp_*) contributed to increasing Δ*P_max_* linearly.

Second, to induce RBC sedimentation in a driving syringe, test blood (Hct = 50%) was prepared by suspending normal RBCs into dextran solution (20 mg/mL). Herein, RBC sedimentation occurred during two steps of blood loading (i.e., air compression-based blood loading: RBC aggregation quantification, and syringe pump-based blood loading: blood viscosity quantification). As depicted in Figure 5B(i), time-resolved *Q_mc_* and Δ*P* were obtained with respect to *Q_sp_* = 4 and 10 mL/h. Interestingly, the plateau value of Δ*P* exhibited a small increase as the *Q_sp_* was increased from 4 mL/h to 10 mL/h. As shown in Figure 5B(ii), by varying *Q_sp_* ranging from 4 mL/h to 10 mL/h, blood viscosity (*μ_b_*) was plotted as a function of *Q_mc_*. With the exception of *Q_sp_* = 4 mL/h, the *μ_b_* remained unchanged with respect to *Q_mc_*. The corresponding blood viscosity of each *Q_sp_* was obtained as *μ_b_* = 6.16 ± 0.37 cP (*n* = 303) for *Q_sp_* = 4 mL/h, *μ_b_* = 4.46 ± 0.17 cP (*n* = 326) for *Q_sp_* = 6 mL/h, *μ_b_* = 3.30 ± 0.09 cP (*n* = 299) for *Q_sp_* = 8 mL/h, and *μ_b_* = 2.32 ± 0.08 cP (*n* = 247) for *Q_sp_* = 10 mL/h. Notably, the *μ_b_* increased markedly when *Q_sp_* was reduced from 10 mL/h to 4 mL/h. As shown in Figure 5B(iii), variations of *μ_b_* and Δ*P_max_* were obtained with respect to *Q_sp_* = 2, 4, 6, 8, and 10 mL/h. With regard to *μ_b_*, when *Q_sp_* exceeded 4 mL/h, increasing *Q_sp_* caused a reduction in blood viscosity, suggesting that RBC sedimentation became pronounced at a lower value of *Q_sp_*. Furthermore, the Δ*P_max_* increased gradually for up to *Q_sp_* = 6 mL/h. For *Q_sp_* > 6 mL/h, the Δ*P_max_* showed no appreciable change with respect to *Q_sp_*. Because blood viscosity decreased substantially at higher flow rates, the Δ*P_max_* remained constant within the specific range of *Q_sp_*. Compared with results for control blood (Figure 5A(iii)), the RBC sedimentation in the driving syringe contributed to altered values of *μ_b_* and Δ*P_max_*.

Third, to compare with the results obtained by the proposed method, the viscosities of control blood and test blood were measured with the coflowing streams [1,82,99,100]. The previous method [101] indicates that the microfluidic method (i.e., coflowing streams method) provides consistent results when compared with the conventional cone-and-plate viscometer. Thus, the previous method was employed to adapt and validate the viscosity results obtained by the present method. In contrast to the proposed method, the previous approach measured blood viscosity without quantifying RBC aggregation. Therefore, RBC sedimentation occurred only during blood delivery. Figure 5C(i) depicts coflowing streams for the measurement of blood viscosity. Herein, the flow rate of test blood is set to *Q_b_* = 10 mL/h. To shift the interface near channel center (i.e., *β* = *W_b_*/*W* = 0.52), the flow rate of reference fluid was adjusted to *Q_r_* = 35 mL/h. Considering that both streams had the same pressure drop in a single channel, the formula of blood viscosity (*μ_b_*) was given as $\mu_{b}=\mu_{r}\times \left(\frac{\beta }{1-\beta }\right)\times \left(\frac{Q_{r}}{Q_{b}}\right)$. Herein, the *μ_r_* denoted viscosity of the reference fluid (1× PBS). As shown in Figure 5C(ii), temporal variations of *μ_b_* were obtained with respect to blood medium (i.e., 1× PBS, and dextran sol. [20 mg/mL]) and blood flow rate (i.e., *Q_b_* = 1, and 10 mL/h). With regard to control blood (1× PBS, Hct = 50%), the corresponding viscosity of each blood flow rate was obtained as *μ_b_* = 3.71 ± 0.04 cP (*n* = 411) for *Q_sp_* = 1 mL/h, and *μ_b_* = 2.85 ± 0.02 cP (*n* = 626) for *Q_sp_* = 10 mL/h. Additionally, for test blood (i.e., dextran sol. [20 mg/mL], Hct = 50%), the corresponding viscosity of each blood flow rate was measured as *μ_b_* = 5.36 ± 0.12 cP (*n* = 2234) for *Q_sp_* = 1 mL/h, and *μ_b_* = 4.89 ± 0.04 cP (*n* = 302) for *Q_sp_* = 10 mL/h. As shown in Figure 5C(iii), for different bloods (i.e., control blood and test blood), the *μ_b_* was plotted as a function of blood flow rate (*Q_b_*). From the results, blood viscosity tended to decrease gradually when *Q_b_* was increased from 2 mL/h to 6 mL/h. Above *Q_b_* = 6 mL/h, blood viscosity remained unchanged with respect to *Q_b_*. With regard to the blood medium, dextran solution (20 mg/mL) increased blood viscosity markedly when compared with 1× PBS.

At last, for two bloods (i.e., control blood and test blood), blood viscosity obtained by both methods (i.e., proposed method: pro. m., previous method: prev. m.) was compared quantitatively. As shown in Figure 5D(i), with regard to control blood, blood viscosity (*μ_b_*) obtained by both methods was plotted as a function of delivered blood flow rate (*Q_sp_*). In the inset, a scatter plot was drawn to represent the linear correlation between blood viscosities measured using both methods. The regression formula was obtained as *μ_b_* (pro. m.) = 0.6778*μ_b_* (prev. m.) + 1.6581 (R^2^ = 0.7235). Because the linear regression yielded a high value of R^2^, blood viscosity obtained by both methods could be considered comparable. Similarly, as shown in Figure 5D(ii), with respect to test blood, blood viscosity (*μ_b_*) obtained by both methods was plotted as a function of *Q_sp_*. As shown in the inset, a scatter plot was drawn to indicate the linear relationship between blood viscosities obtained by both methods. According to linear regression analysis, a linear regression curve was obtained as *μ_b_* (pro. m.) = 11.396*μ_b_* (prev. m.) − 51.72 (R^2^ = 0.6193). In particular, the blood flow rate of the syringe pump had a strong influence on blood viscosity. As shown in Figure 5D(i), for control blood, both methods gave comparable viscosity. Nonetheless, for test blood, both methods exhibited a moderate correction with respect to *Q_sp_*. In the previous approach, blood viscosity was quantified without incorporating an RBC aggregation assessment. As a result, the previous method did not require the ~120 s aggregation quantification interval, thereby minimizing the possibility of RBC sedimentation in the driving syringe. It can be inferred that sedimentation during the aggregation quantification step contributed to variations in the measured viscosity. Moreover, blood viscosity determined by the proposed method was highly dependent on the delivered blood flow rate. As shown un in Figure 5C(iii), the previous method yielded nearly constant viscosity values when *Q_sp_* exceeded 6 mL/h. Therefore, the *Q_sp_* should be maintained above 6 mL/h for ensuring flow rate-independent viscosity. In addition, to reduce large fluctuations resulting from RBC sedimentation in the driving syringe, the blood flow rate should be set to the highest feasible level. Unless otherwise specified, in the subsequent experiments, for convenience, the blood flow rate was set to *Q_sp_* = 10 mL/h.

### 3.4. Contribution of Hematocrit (Hct)

Because hematocrit strongly influences RBC aggregation and blood viscosity, the hematocrit of test blood is typically adjusted to a specified value [58,89,102]. In this subsection, the contribution of hematocrit was validated using the proposed method. Herein, to induce RBC aggregation, the concentration of dextran solution (20 mg/mL) was selected as the blood medium. Hematocrit of test blood was then adjusted to Hct = 30~60% by suspending normal RBCs in the specific dextran solution. Blood (*V_b_* = 200 μL) was loaded into a driving syringe.

**Figure 6:**
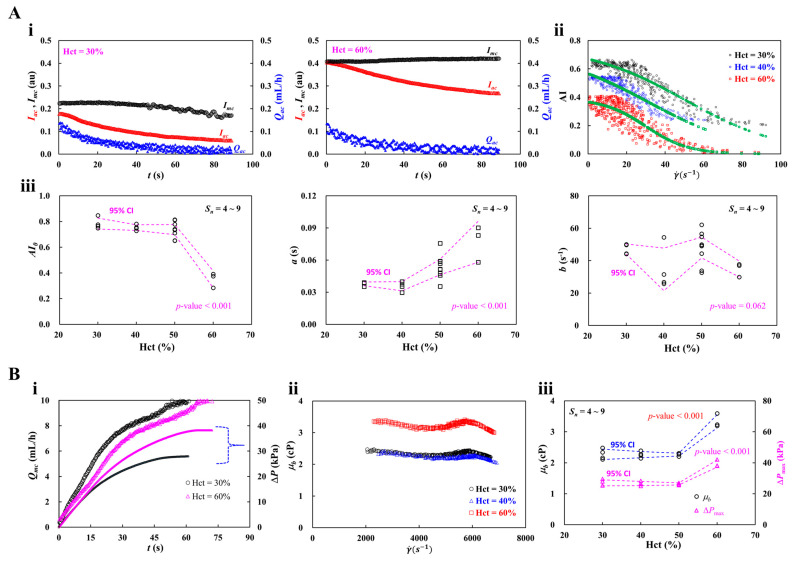
Contribution of hematocrit to RBC aggregation and blood viscosity obtained by the proposed method. Herein, the hematocrit of the test blood was adjusted to Hct = 30~60% by suspending normal RBCs into dextran solution (20 mg/mL). Blood (200 μL) was loaded into a driving syringe. Flow rate of syringe pump set to *Q_sp_* = 10 mL/h. (**A**) Quantification of RBC aggregation with respect to hematocrit. (**i**) Temporal variations of *I_mc_*, *I_ac_*, and *Q_ac_* with respect to Hct = 30% and 60%. (**ii**) Shear rate ($\dot{\gamma }$)-dependent AI with respect to Hct = 30%, 40%, and 60%. Based on the specified regression formula (i.e., AI = $\frac{{AI}_{0}}{1+exp(a\left[\dot{\gamma }-b\right])}$), the corresponding regression formula of each hematocrit was obtained as AI = 0.7649/(1 + exp [0.039 ($\dot{\gamma }\;$ − 50.0872)]) for Hct = 30%, AI = 0.7275/(1 + exp [0.0398 ($\dot{\gamma }$ − 31.5752)]) for Hct = 40%, and AI = 0.3914/(1 + exp [0.0902 ($\dot{\gamma }$ − 29.8718)]) for Hct = 60%. (**iii**) Variations of three parameters (i.e., *AI*_0_, *a*, and *b*) obtained by conducting regression analysis with respect to Hct. (**B**) Contribution of hematocrit to blood viscosity. (**i**) Time-dependent *Q_mc_* and Δ*P* with respect to Hct = 30% and 60%. (**ii**) Variations of *μ_b_* with respect to $\dot{\gamma }$. (**iii**) Variations of *μ_b_* and Δ*P_max_* with respect to Hct.

First, to quantify RBC aggregation, as shown in Figure 6A(i), time-lapse *I_mc_*, *I_ac_*, and *Q_ac_* were plotted with respect to Hct = 30% and 60%. RBC aggregation index (AI) was calculated using time-lapse *I_mc_* and *I_ac_*. Simultaneously, shear rate ($\dot{\gamma }$) was estimated by substituting time-resolved *Q_ac_* into the shear rate formula. As shown in Figure 6A(ii), by varying hematocrit ranging from Hct = 30% to Hct = 60%, the AI was plotted as a function of $\dot{\gamma }$. The shear-dependent AI was best fitted as AI = $\frac{{AI}_{0}}{1+exp(a\left[\dot{\gamma }-b\right])}$. The green dashed line represented the best-fitting regression curve. The corresponding curve-fitting formula of each hematocrit was obtained as AI = 0.7649/(1 + exp [0.039($\dot{\gamma }\;$ − 50.0872)]) for Hct = 30%, AI = 0.7275/(1 + exp [0.0398($\dot{\gamma }$ − 31.5752)]) for Hct = 40%, and AI = 0.3914 / (1 + exp [0.0902($\dot{\gamma }$ − 29.8718)]) for Hct = 60%. Based on three parameters (i.e., *AI*_0_, *a*, and *b*) estimated by conducting non-linear regression analysis, as shown in Figure 6A(iii), variations of three parameters were represented with respect to Hct = 30~60%. Herein, the number of test blood was set to *S_n_* = 4~9. Based on a statistical test (i.e., one-way ANOVA), the corresponding *p*-value of each parameter was obtained as *p*-value < 0.001 for parameter *AI*_0_, *p*-value < 0.001 for parameter *a*, and *p*-value = 0.062 for parameter *b*. The *AI*_0_ decreased slightly from Hct = 30% to Hct = 50%, but declined markedly between Hct = 50% and Hct = 60%. The parameter *a* was unchanged between Hct = 30% and 40%, but increased gradually from Hct = 40% to Hct = 60%. The parameter *b* did not show a clear trend because it exhibited large scatter with respect to Hct. However, the *b* showed a substantial difference between Hct = 30% and Hct = 60%. Given that the conventional RBC aggregation index showed substantial hematocrit-dependent variation [16,103], the parameters obtained using the proposed method (i.e., *AI*_0_ and *a*) could be used as promising indices.

Second, after RBC aggregation quantification, the contribution of hematocrit to blood viscosity was assessed using the proposed method. As shown in Figure 6B(i), time-dependent *Q_mc_* and Δ*P* were obtained with respect to Hct = 30% and 60%. The rising time increased markedly as hematocrit rose from Hct = 30% to Hct = 60%. In addition, the plateau value of Δ*P* increased substantially at the highest hematocrit. Figure 6B(ii) showed variations of *μ_b_* with respect to shear rate ($\dot{\gamma }$). The *μ_b_* did not exhibit substantial variation with respect to shear rate. The corresponding viscosity of each hematocrit was summarized as *μ_b_* = 2.34 ± 0.06 cP (*n* = 208) for Hct = 30%, *μ_b_* = 2.24 ± 0.06 cP (*n* = 199) for Hct = 40%, and *μ_b_* = 3.23 ± 0.10 cP (*n* = 233) for Hct = 60%. That is, blood viscosity showed no substantial difference between Hct = 30% and Hct = 40%. However, blood viscosity increased significantly as hematocrit increased from Hct = 40% to Hct = 60%.

As shown in Figure 6B(iii), variations of *μ_b_* and Δ*P_max_* were plotted as a function of hematocrit. According to a statistical test (i.e., one-way ANOVA), the *p*-value was less than 0.001 for both properties. According to the previous studies [58,89,104], hematocrit contributed to increasing blood viscosity significantly. However, both properties remained unchanged from Hct = 30% and Hct = 50%, but increased markedly from Hct = 50% to Hct = 60%. In contrast to the previous methods, the present method quantified RBC aggregation before measuring blood viscosity. During aggregation quantification, RBC sedimentation inevitably occurred in the driving syringe while blood was delivered at a low flow rate by air compression. In addition, the dextran solution (20 mg/mL) markedly accelerated sedimentation in the syringe. This phenomenon was expected to be more pronounced at low hematocrit (Hct = 30% or 40%) [105]. After RBC aggregation quantification, when the test blood with low hematocrit (i.e., Hct = 30~50%) was introduced into the microfluidic channels, the allocated hematocrit exhibited no substantial difference. Consequently, blood viscosity was inferred to show little difference over Hct = 30~50%.

From the experimental investigation, the RBC aggregation index (AI) exhibited greater sensitivity to hematocrit variations than blood viscosity. Interestingly, owing to RBC sedimentation in a driving syringe, blood viscosity showed no substantial difference among low-hematocrit blood (i.e., Hct < 50%).

### 3.5. Contribution of Blood Medium (Dextran Concentration)

According to previous studies, the autologous plasma (i.e., plasma proteins) strongly affects RBC aggregation and blood viscosity [58,89,106,107]. Instead of diluting autologous plasma, diluted dextran solutions have been widely used as a standard aggregating medium and can increase blood viscosity [108,109,110,111]. According to the previous study [105], since physiological fibrinogen concentration is typically under 4 mg/mL, increasing fibrinogen from 4 mg/mL to 8 mg/mL led to roughly a twofold rise in aggregation indices. Adding dextran solution (10 mg/mL) into normal RBC suspensions increased the indices more than threefold when compared with 8 mg/mL fibrinogen. The indices increased significantly for up to 40 mg/mL. For this reason, the maximum value of concentration of dextran solution (i.e., 20 mg/mL) stimulated RBC aggregation strongly when compared with the normal level of fibrinogen. The maximum concentration of dextran solution was limited to 20 mg/mL. In this subsection, to quantify the effect of blood medium, test blood (Hct = 50%) was prepared by suspending normal RBCs in dextran solution (*C_dex_* = 0, 5, 10, 15, and 20 mg/mL). The *C_dex_* = 0 denoted pure 1× PBS. Blood (200 μL) was loaded into a driving syringe.

**Figure 7:**
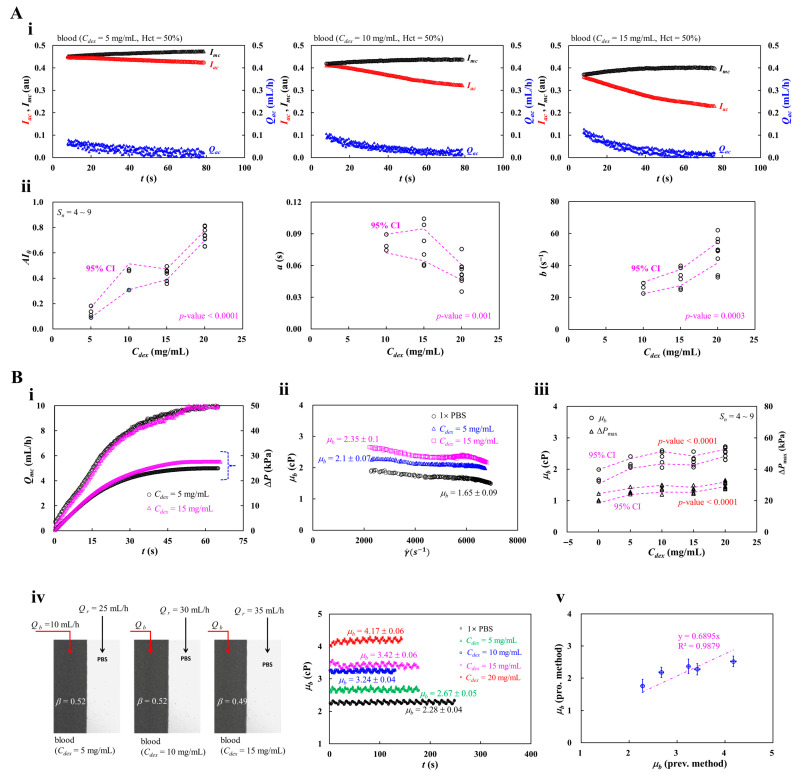
Contribution of blood medium (i.e., dextran solution) to RBC aggregation and blood viscosity. To quantify the effect of blood medium, test blood (Hct = 50%) was prepared by adding normal RBCs into dextran solution (*C_dex_* = 0, 5, 10, 15, and 20 mg/mL). (**A**) Contribution of dextran solution to RBC aggregation. (**i**) Time-lapse *I_mc_*, *I_ac_*, and *Q_ac_* with respect to *C_dex_* = 5, 10, and 15 mg/mL. (**ii**) Variations of three parameters obtained by regression analysis with respect to *C_dex_*. (**B**) Contribution of dextran solution to blood viscosity. (**i**) Time-dependent *Q_mc_* and Δ*P* with respect to *C_dex_* = 5 and 15 mg/mL. (**ii**) Variations of *μ_b_* with respect to shear rate ($\dot{\gamma }$). (**iii**) Variations of *μ_b_* and Δ*P_max_* with respect to *C_dex_*. (**iv**) Blood viscosity obtained by the previous method (i.e., coflowing streams method). The left-side panel showed microscopic images for quantifying blood viscosity. The right-side panel showed temporal variations of *μ_b_* with respect to *C_dex_* = 0~20 mg/mL. (**v**) Quantitative comparison between blood viscosity obtained by both methods. According to linear regression analysis, the regression formula was obtained as *μ_b_* (pro. m.) = 0.6895*μ_b_* (prev. m.) (R^2^ = 0.9879).

First, to quantify RBC aggregation under different blood media, as shown in Figure 7A(i), time-lapse *I_mc_*, *I_ac_*, and *Q_ac_* were obtained with respect to *C_dex_* = 5, 10, and 15 mg/mL. According to the results, the intensity difference (i.e., Δ*I* = *I_mc_* − *I_ac_*) increased substantially when concentrations of dextran solution increased from 5 mg/mL to 15 mg/mL. Based on the regression model of the AI, three parameters (i.e., *AI*_0_, *a*, and *b*) were obtained by conducting non-linear regression analysis. Figure 7A(ii) depicts variations of three parameters with respect to *C_dex_*. The number of control blood samples was set to *S_n_* = 4~9. Since the AI variation at *C_dex_* = 5 was not well fitted, the corresponding parameters (*a*, *b*) were excluded from the scatter plot. According to a statistical test (i.e., one-way ANOVA), the corresponding *p*-value of each parameter was obtained as *p*-value < 0.0001 for parameter *AI*_0_, *p*-value = 0.001 for parameter *a*, and *p*-value = 0.0003 for parameter *b*. From the results, three parameters exhibited substantial differences with respect to *C_dex_*. The previous studies also reported that the conventional aggregation index exhibited a substantial difference with respect to the specific concentration of dextran solution [89,109]. From the experimental investigation, three parameters obtained by the proposed method could be used effectively as promising indices for quantifying RBC aggregation.

Second, the present method was employed to measure blood viscosity with respect to *C_dex_*. As shown in Figure 7B(i), time-dependent *Q_mc_* and Δ*P* were obtained with respect to *C_dex_* = 5 and 15 mg/mL. The rising time of *Q_mc_* and the plateau value of the Δ*P* were increased significantly when the dextran concentration was increased from 5 mg/mL to 15 mg/mL. As shown in Figure 7B(ii), by changing the concentration of dextran solution ranging from *C_dex_* = 0 to *C_dex_* = 15 mg/mL, the *μ_b_* was plotted as a function of $\dot{\gamma }$. From the results, blood viscosity tended to decrease slightly with respect to shear rate. A pronounced increase in blood viscosity was observed at higher concentrations. Instead of conducting a best-fitted curve, blood viscosity values were summarized as mean ± standard deviation (*n*: number of data points). The corresponding viscosity of each *C_dex_* was estimated as *μ_b_* = 1.65 ± 0.09 cP (*n* = 218) for 1× PBS, *μ_b_* = 2.1 ± 0.07 cP (*n* = 221) for *C_dex_* = 5 mg/mL, and *μ_b_* = 2.35 ± 0.1 cP (*n* = 221) for *C_dex_* = 15 mg/mL. Figure 7B(iii) showed variations of *μ_b_* and Δ*P_max_* with respect to *C_dex_*. The number of test blood was set to *S_n_* = 4~9. According to a statistical test (i.e., one-way ANOVA), the corresponding *p*-value of both parameters was less than 0.0001. The results indicated that the dextran solution contributed to increasing *μ_b_* and Δ*P_max_* markedly. To quantitatively compare with blood viscosity values obtained by the proposed method, as shown in Figure 7B(iv), the previous method was applied to measure the blood viscosity of the same test bloods. Herein, as the previous method was only aimed at measuring blood viscosity, it excluded RBC aggregation quantification.

In particular, from the perspective of the proposed method, the effect of RBC sedimentation during the blood-delivery step used for aggregation quantification was eliminated. The left-side panel showed microscopic images for quantifying blood viscosity. Herein, the flow rate of the test blood is set to *Q_b_* = 10 mL/h. To maintain an interface between the test blood and reference fluid (1× PBS) near the channel center, flow rate of reference fluid was adjusted as *Q_r_* = 25 mL/h for *C_dex_* = 5 mg/mL, *Q_r_* = 30 mL/h for *C_dex_* = 10 mg/mL, and *Q_r_* = 35 mL/h for *C_dex_* = 15 mg/mL. Herein, the corresponding interface of each test blood was calculated as *β* = 0.52 for *C_dex_* = 5 mg/mL, *β* = 0.52 for *C_dex_* = 10 mg/mL, and *β* = 0.49 for *C_dex_* = 15 mg/mL. The right-side panel showed temporal variations of *μ_b_* with respect to *C_dex_* = 0~20 mg/mL. As expected, blood viscosity increased markedly when the dextran concentration increased from *C_dex_* = 0 to *C_dex_* = 20 mg/mL. The corresponding viscosity of each concentration of dextran solution was obtained as *μ_b_* = 2.28 ± 0.04 cP (*n* = 494) for 1× PBS, *μ_b_* = 2.67 ± 0.05 cP (*n* = 355) for *C_dex_* = 5 mg/mL, *μ_b_* = 3.24 ± 0.04 cP (*n* = 261) for *C_dex_* = 10 mg/mL, *μ_b_* = 3.42 ± 0.06 cP (*n* = 354) for *C_dex_* = 15 mg/mL, and *μ_b_* = 4.17 ± 0.06 cP (*n* = 288) for *C_dex_* = 20 mg/mL. To quantitatively compare with viscosity values obtained by both methods, as shown in Figure 7B(v), a scatter plot was created by plotting *μ_b_* (prev. m.) along the horizontal axis and *μ_b_* (pro. m.) along the vertical axis. A linear regression analysis was performed to probe the correlation between both methods. According to linear regression analysis, the regression formula was obtained as *μ_b_* (pro. m.) = 0.6895*μ_b_* (prev. m.) (R^2^ = 0.9879). As the regression coefficient was calculated as a higher value of R^2^ = 0.9879, viscosity values obtained by both methods could be regarded as comparable and exhibited strong linearity.

From the experimental investigation, the proposed method was successfully employed to prove the contribution of blood medium (i.e., dextran solution) to RBC aggregation and blood viscosity. Specifically, the proposed method had the ability to provide consistent results compared with the previous method.

### 3.6. Contribution of Blood-Loading Volume (V_b_) into a Driving Syringe

**Figure 8:**
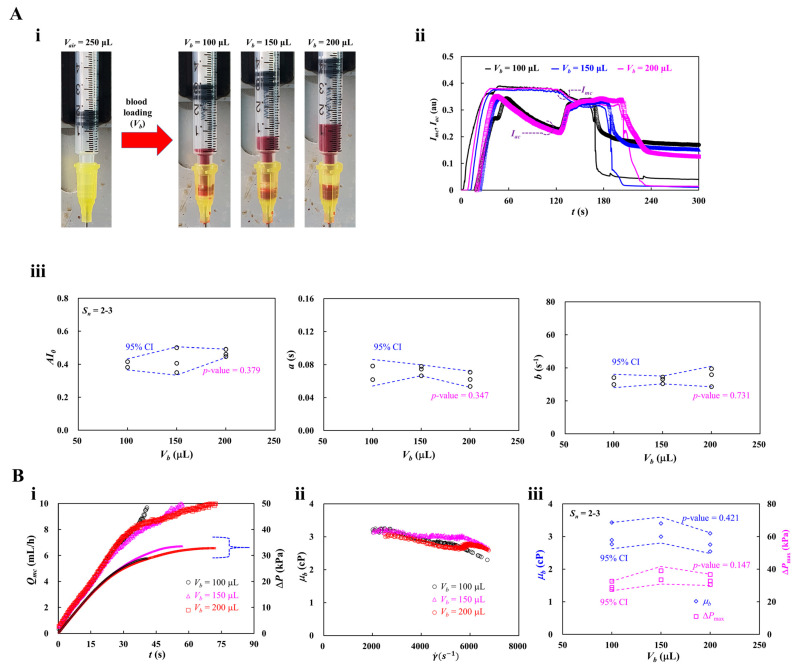
Contribution of blood-loading volume to RBC aggregation and blood viscosity. Test blood (Hct = 50%) was prepared by suspending normal RBCs into dextran solution (20 mg/mL). (**A**) Contribution of blood-loading volume (*V_b_* = 100, 150, and 200 μL) to RBC aggregation. (**i**) Snapshot for showing blood volume (*V*_b_) supplied into a driving syringe. (**ii**) Time-lapse *I_mc_* and *I_ac_* with respect to *V_b_*. (**iii**) Variations of three parameters (i.e., *AI*_0_, *a*, and *b*) with respect to *V_b_*. (**B**) Contribution of blood-loading volume (*V_b_*) to blood viscosity. (**i**) Time-lapse *Q_mc_* and Δ*P* with respect to *V_b_*. (**ii**) Variations of *μ_b_* with respect to $\dot{\gamma }$. (**iii**) Variations of *μ_b_* and Δ*P_max_* with respect to *V_b_*.

For all experiments, 200 μL of blood was loaded into a driving syringe. As shown in Figure 8A(i), an air cavity of 250 μL was secured in the syringe, after which the blood volume (*V_b_*) was suctioned. In the two-step delivery procedure, the air cavity was first compressed by approximately 50 μL, which drove blood from the syringe into the microfluidic channels. This initial air-driven step also removed pre-existing air bubbles in the fluidic path, resulting in complete blood-filling of the channels. Once 50 μL of blood was discharged, delivery was stopped immediately. Subsequently, the remaining blood (~150 μL) was infused into the microfluidic channels using a syringe pump. Notably, about 150 μL of blood was still considered a sufficiently large volume for blood viscosity measurements. In this study, the blood flow rate was calibrated using the steady-state blood velocity. Due to the air compliance effect in the syringe, blood velocity did not reach its plateau immediately after the syringe pump was activated. Therefore, blood had to be delivered continuously until the velocity stabilized at the plateau. Accordingly, this subsection aimed to determine the minimum blood-loading volume required to ensure reliable blood viscosity measurements. Test blood (Hct = 50%) was prepared by suspending normal RBCs into dextran solution (20 mg/mL). As shown in Figure 8A(i), the blood-loading volume was set to *V_b_* = 100, 150, and 200 μL.

Figure 8A(ii) showed time-lapse *I_mc_* and *I_ac_* with respect to *V_b_*. As expected, *I_mc_* and *I_ac_* did not exhibit notable changes with respect to *V_b_* during air-compression delivery. However, during syringe pump delivery, the plateau periods of *I_mc_* and *I_ac_* increased as the *V_b_* increased. As shown in Figure 8A(iii), variations of three parameters (i.e., *AI*_0_, *a*, and *b*) were obtained with respect to *V_b_*. The number of test blood was set to *S_n_* = 2~3. According to a statistical test (i.e., one-way ANOVA), the corresponding *p*-value of each parameter was obtained as *p*-value = 0.379 for parameter *AI*_0_, *p*-value = 0.347 for parameter *a*, and *p*-value = 0.731 for parameter *b*. The results indicated that the specified ranges of blood-loading volume did not significantly affect RBC aggregation.

As shown in Figure 8B(i), time-lapse *Q_mc_* and Δ*P* were obtained with respect to *V_b_*. Increasing *V_b_* led to a longer rising time of *Q_mc_*. When *V_b_* was set to 100 μL, the steady-state Δ*P* was reduced compared with the other conditions. However, the steady plateau of Δ*P* remained unchanged as *V_b_* increased from 150 μL to 200 μL. Figure 8B(ii) depicts variations of *μ_b_* with respect to $\dot{\gamma }$. The correspond viscosity of each *V_b_* was obtained as *μ_b_* = 2.89 ± 0.22 cP (*n* = 119) for *V_b_* = 100 μL, *μ_b_* = 3.00 ± 0.12 cP (*n* = 182) for *V_b_* = 150 μL, and *μ_b_* = 2.75 ± 0.11 cP (*n* = 232) for *V_b_* = 200 μL. As shown in Figure 8B(iii), variations of *μ_b_* and Δ*P_max_* were obtained with respect to *V_b_*. According to a statistical test (i.e., one-way ANOVA), the corresponding *p*-value of each parameter was obtained as *p*-value = 0.421 for *μ_b_*, and *p*-value = 0.147 for Δ*P_max_*. The results indicated that consistent blood viscosity measurement was achievable with blood-loading volumes from 100 μL to 200 μL.

From the experimental measurements, it was confirmed that at least 100 μL of blood was required to ensure consistent results.

### 3.7. Detection of Heat-Shocked RBCs

According to earlier studies [85,106,112,113,114], when normal RBCs are subjected to elevated temperatures exceeding 40 °C, both blood viscosity and RBC aggregation differ substantially from those under the normal physiological temperature of 37 °C. In this subsection, the proposed method was employed to probe the contribution of heat-shocked RBCs to blood viscosity and RBC aggregation. Herein, the exposure time of each temperature is set to 40 min for 45 °C and 20 min for 50 °C. Test blood (Hct = 50%) was prepared by suspending heat-shocked RBCs into dextran solution (20 mg/mL). Blood (*V_b_* = 200 μL) was loaded into a driving syringe.

**Figure 9:**
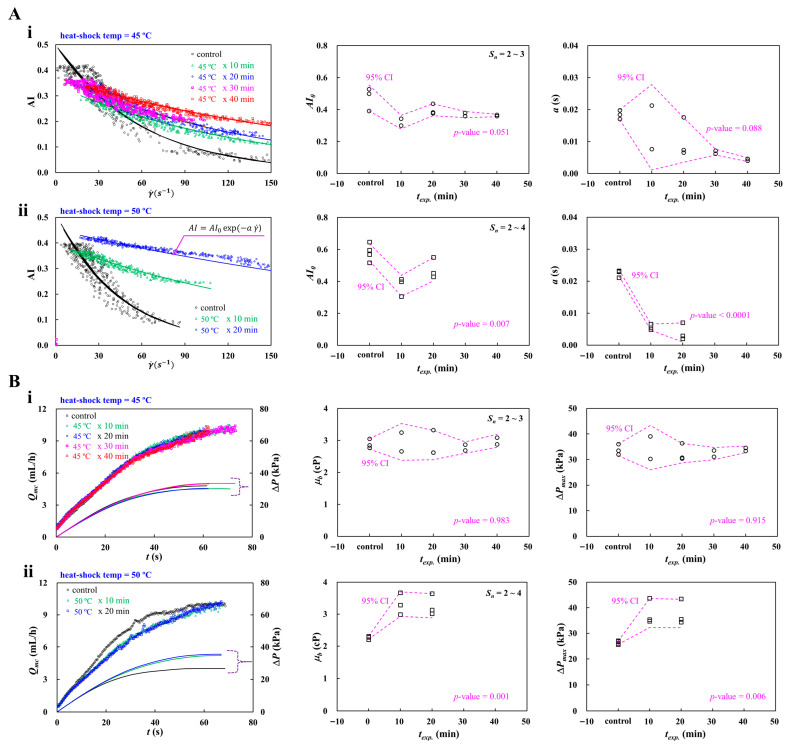
Contribution of heat-shocked RBCs to RBC aggregation and blood viscosity. Herein, the exposure time for each temperature is set to 40 min for 45 °C and 20 min for 50 °C. Test blood (Hct = 50%) was prepared by suspending heat-shocked RBCs into dextran solution (20 mg/mL). (**A**) Contribution of heat-exposed RBCs to RBC aggregation. (**i**) Quantification of RBC aggregation using RBCs exposed to 45 °C for up to 40 min. The first panel showed variations of AI with respect to $\dot{\gamma }$. The AI was best fitted as AI = *AI*_0_ exp (−*a* $\dot{\gamma }$). The second panel showed variations of parameter *AI*_0_ with respect to exposure time (*t_exp_*). The last panel showed variations of parameter *a* with respect to *t_exp_*. (**ii**) Quantification of RBC aggregation using RBCs exposed to 50 °C for up to 20 min. The first panel showed variations of AI with respect to $\dot{\gamma }$. The second panel showed variations of parameter *AI*_0_ with respect to exposure time (*t_exp_*). The last panel showed variations of parameter *a* with respect to *t_exp_*. (**B**) Contribution of heat-shocked RBCs to blood viscosity. (**i**) Quantification of blood viscosity for RBCs exposed to 45 °C for up to 40 min. The first panel showed time-lapse *Q_mc_* and Δ*P*. The second panel showed variations of *μ_b_* with respect to *t_exp_*. The last panel showed Δ*P_max_* with respect to *t_exp_*. (**ii**) Quantification of blood viscosity for RBCs exposed to 50 °C for up to 20 min. The first panel showed time-lapse *Q_mc_* and Δ*P*. The second panel showed variations of *μ_b_* with respect to *t_exp_*. The last panel showed Δ*P_max_* with respect to *t_exp_*.

First, variations of the RBC aggregation index (AI) were quantitatively assessed as a function of thermal exposure conditions. As shown in Figure 9A(i), RBC aggregation was quantified for RBCs exposed to 45 °C for up to 40 min. The first panel showed variations of AI with respect to $\dot{\gamma }$. As the thermal exposure duration increased, the variation of AI decreased significantly. Herein, as variations of AI were not well represented by a sigmodal function, the regression formula of AI was replaced by AI = *AI*_0_ exp (−*a* $\dot{\gamma }$). Two unknown parameters (i.e., *AI*_0_, and *a*) were then obtained by conducting non-linear regression analysis. The second panel showed variations of parameter *AI*_0_ with respect to thermal exposure time (*t_exp_*). The number of test blood was set to *S_n_* = 2~3. The statistical test (i.e., one-way ANOVA) indicated a *p*-value of 0.051. Except for *t_exp_* = 10 min, the *AI*_0_ did not exhibit statistical significance with respect to *t_exp_*. The last panel showed variations of parameter *a* with respect to *t_exp_*. According to a statistical test (i.e., one-way ANOVA), the *p*-value of parameter *a* was obtained as 0.088. As exposure time became longer, the overall magnitude of parameter *a* decreased progressively. As depicted in Figure 9A(ii), RBC aggregation was quantified for RBCs exposed to 50 °C for up to 20 min. The first panel showed variations of AI with respect to $\dot{\gamma }$. A significant reduction in AI variation was observed with longer exposure times. The second panel showed variations of parameter *AI*_0_ with respect to *t_exp_*. The one-way ANOVA gave a *p*-value of 0.007. The number of test blood was set to *S_n_* = 2~4. In comparison with *AI*_0_ obtained at 45 °C (Figure 9A(i)), the *AI*_0_ exhibited large fluctuations. The last panel showed variations of parameter *a* with respect to *t_exp_*. According to a statistical test (i.e., one-way ANOVA test), the *p*-value was obtained as less than 0.0001. The parameter *a* decreased significantly as exposure time increased. From the results, the parameter *a* changed substantially when normal RBCs were exposed to higher temperatures and for longer durations.

Second, blood viscosity was assessed for RBCs exposed to specific temperatures and durations. Figure 9B(i) showed quantification of blood viscosity for RBCs exposed to 45 °C for up to 40 min. The first panel showed time-lapse *Q_mc_* and Δ*P* with respect to exposure time. The second panel showed variations of *μ_b_* with respect to *t_exp_*. According to a statistical test (i.e., one-way ANOVA), a *p*-value was obtained as 0.983. From the results, no significant change in blood viscosity was observed after exposing RBCs to 45 °C for up to 40 min. The last panel showed variations of Δ*P_max_* with respect to *t_exp_*. According to the statistical test (i.e., one-way ANOVA), the *p*-value was obtained as 0.915. The Δ*P_max_* did not exhibit a statistically meaningful difference under 45 °C exposure for as long as 40 min.

Figure 9B(ii) depicts the quantification of blood viscosity for RBCs exposed to 50 °C for up to 20 min. The first panel showed time-lapse *Q_mc_* and Δ*P* with respect to exposure time. When RBCs were exposed to 50 °C for 10 min, the rising time of *Q_mc_* increased, and the steady plateau of Δ*P* became higher. No additional substantial changes were observed when the exposure duration was extended from 10 to 20 min. The second panel showed variations of *μ_b_* with respect to *t_exp_*. The statistical test (i.e., one-way ANOVA) gave *p*-value = 0.001. A marked difference in blood viscosity was observed for RBCs exposed to 50 °C for 10 min, whereas no statistically significant change occurred when the exposure duration was increased from 10 min to 20 min. The last panel showed Δ*P_max_* with respect to *t_exp_*. The pattern of Δ*P_max_* was very similar to that of blood viscosity. The statistical test (i.e., one-way ANOVA) resulted in statistical significance (*p*-value = 0.006). Experimental results indicated that RBC structural integrity was preserved after exposure to 45 °C for up to 40 min [115,116]. In contrast, exposure to 50 °C for 10 min may have induced structural damage [115,117,118], which was accompanied by an increase in blood viscosity (*μ_b_*) and maximum pressure difference (Δ*P_max_*) [90,119].

From the experimental investigation, with regard to RBCs exposed to 45 °C, RBC aggregation exhibited a substantial difference. However, no significant difference in blood viscosity was observed. Thus, it was confirmed that the RBC aggregation index could be used effectively for monitoring thermal-induced damage of RBCs.

As a limitation, the proposed method could not measure RBC aggregation and blood viscosity simultaneously. During the time interval (~120 s) required for RBC aggregation quantification, RBC sedimentation occurred, which impeded the subsequent viscosity determination. Further methodological improvements are therefore needed to allow both parameters to be measured concurrently. In addition, the proposed hybrid delivery system introduced operator-dependent variability and limited high-throughput usage in clinical settings. Future work will focus on developing a novel method to address these technical issues.

## 4. Conclusions

In this study, a novel method was demonstrated to resolve several issues (i.e., dead volume loss in the fluid path, hematocrit-sensitive blood velocity calibration, RBC sedimentation in a driving syringe, and flow-dependent hemorheological properties) raised by the previous methods. First, to reduce dead volume loss in the fluidic path (i.e., syringe needle, inlet tubing, and channels), an air cavity (*V_air_* = 250 μL) was secured above the blood column (at least 100 μL) loaded into a driving syringe. Second, to calibrate hematocrit-sensitive blood velocity fields and minimize RBC sedimentation in a driving syringe, a single syringe pump was set to a higher value of flow rate (*Q_sp_* = 10 mL/h). Third, to probe flow-dependent RBC aggregation and blood viscosity sequentially, a microfluidic channel was carefully designed to have a main channel (i.e., high shear rate: $\dot{\gamma }$ > 1000 s^−1^) and an aggregation channel (i.e., low shear rate: $\dot{\gamma }$ < 50 s^−1^). RBC aggregation index (AI) was then assessed by comparing the image intensity of blood flow in both channels, especially under air-compression delivery. Shear rate-dependent AI was quantitatively analyzed by conducting non-linear regression fitting. A micro-PIV technique was used to obtain the blood flow rate in each channel (i.e., *Q_mc_* for the main channel and *Q_ac_* for the aggregation channel), where the blood flow rate was maintained constant. Both flow rates were obtained accurately by calibrating velocity fields in terms of *Q_sp_* and the plateau value of blood velocity. Next, the viscosity formula was derived by constructing a fluidic circuit model. Air pressure difference in a driving syringe was estimated using the ideal-gas law (i.e., pressure difference = fluidic resistance × flow rate) and time-lapse *Q_mc_*. Blood viscosity was then obtained by substituting air pressure difference (Δ*P*), *Q_mc_*, and *Q_ac_* into the blood viscosity formula. To validate the performance of the proposed method, first, the measurement accuracy of fluid viscosity was validated with glycerin solution (*C_gl_* = 20~50%). The proposed method gave comparable results when compared with reference data. Second, using two kinds of blood medium (i.e., 1× PBS and dextran solution [20 mg/mL]), the effect of RBC sedimentation in the driving syringe was quantified with respect to blood flow rate (*Q_sp_*). RBC sedimentation had a strong impact on blood viscosity rather than RBC aggregation. To minimize the contribution of RBC sedimentation, the blood flow rate set to a higher value of flow rate (*Q_sp_* = 10 mL/h). Third, to probe the effect of RBC volume (i.e., hematocrit) and blood medium (i.e., dextran solution) on RBC aggregation and blood viscosity, test blood was prepared by suspending normal RBCs in dextran solution (i.e., Hct = 30~50%, *C_dex_* = 0~20 mg/mL). RBC aggregation exhibited a substantial difference with respect to hematocrit and dextran concentration. Interestingly, blood viscosity did not show a substantial difference in ranges of hematocrit (Hct = 30~50%) and higher concentration of dextran (above 10 mg/mL), which resulted from RBCs sedimentation in a driving syringe. Fourth, at least 100 μL of blood was required to ensure consistent results of RBC aggregation and blood viscosity. At last, the proposed method was applied to investigate the biomechanical difference in heat-shocked RBCs (i.e., 45 °C for 40 min, and 50 °C for 20 min). RBC aggregation index (AI) was superior to blood viscosity for monitoring thermal-induced damage of RBCs. In conclusion, the suggested method can accurately measure flow-dependent hemorheological properties, where an air cavity (*V_air_* = 250 μL) was secured above the blood column (at least 100 μL) loaded into a driving syringe and syringe pump set to a constant flow rate. Flow-dependent RBC aggregation and blood viscosity could be used to detect substantial changes in RBCs or the blood medium.

## Data Availability

The original contributions presented in this study are included in the article material. Further inquiries can be directed to the corresponding author.
